# The influence of differential leadership and proactive personality on employee in-role performance: An integrated model

**DOI:** 10.3389/fpsyg.2022.978495

**Published:** 2022-12-22

**Authors:** Sze-Ting Chen, Kai Yin Allison Haga

**Affiliations:** ^1^DPU-CIC (Chinese International College - Dhurakij Pundit University), Bangkok, Thailand; ^2^International Master Program in Asia-Pacific Affairs, College of Social Sciences, National Sun Yat-sen University, Kaohsiung, Taiwan

**Keywords:** proactive personality, differential leadership, organizational justice, emotional labor, employee in-role performance

## Abstract

**Purpose:**

Differential leadership does not necessarily bring only negative effects, as it may also become an incentive management strategy. This study explores whether outsiders can actively become insiders through proactive personality traits, or whether they can actively approach resource controllers to remove obstacles at work and enhance their performance in a Chinese cultural setting.

**Methodology:**

A stratified random sampling method was used. The sample objects were medical staff from hospitals in the six urban districts of Beijing. In 2021, a total of 900 online questionnaires were distributed. 524 valid questionnaires were recovered.

**Main findings:**

The results show that differential leadership, defined as favoritism towards insiders and prejudice against outsiders, can cause changes in employees’ sense of organizational justice and in-role performance. Meanwhile, by introducing ‘proactive personality’ as an independent variable that also acts as a moderator, our study confirms that, under differential leadership, employees with a proactive personality can actively improve situational barriers and have better work performance.

**Implications/applications:**

Our research offers managers the following advice: First, it is better to look not only at relationships, but also to understand an employee’s personality characteristics, whether it has a superficial or deep role at work, in order to reduce the turnover rate and to raise productivity. Second, it is important to teach employees to serve customers with a sincere appreciation of their point of view, rather than focusing on presenting an outward appearance of friendliness.

**Novelty/originality:**

This paper contributes to the theory of proactive personality, emotional labor, and differential leadership. Contrary to previous studies, our research has used ‘proactive personality’ as both a distractor and a predictor at the same time. Also, insiders favored by leaders are not found to perform better at work.

## Introduction

Strengthening relationships between differential leadership (DL) and “insiders” and “outsiders” (IO) has become a crucial task for organizations, because high-level talent has become enterprises’ core competitiveness. Good employee performance and attitudes not only help an organization improve efficiency, but also promote the organization’s innovational development and endogenous motivation (Jha and Kumar, 2016). However, due to different cultures and leadership styles, it is inevitable that members, with a large amount of key information and under the action of certain factors, may exhibit negative behaviors in the workplace, thereby affecting their in-role performance (Ghosh et al., 2017). This not only adversely affects the careers of members within the organization, but also harms the organization and damages its legitimate interests. According to a famous Confucius classic, *Book of Rites · Moderation*, the interaction between people should follow the norms of respect and inferiority, closeness and distance, etc. In other words, respect accorded to wise men varies according to different levels and regions, and there is an order of differences even among relatives. To the present day in Chinese social culture, the valuation of these two norms of respect-and-inferiority and of closeness-and-distance, have formed behavior patterns and communication rules (Zheng, 2006).

The importance of DL and IO in an organization has been previously verified, but few studies on these issues have adopted a “proactive personality” perspective. “Proactive personality” in employee performance is particularly important because the leader has the power to promote employees and allocate resources, and because employees must rely on the leader’s instructions to perform their work. Employees in a positive atmosphere have generally good expectations of their leaders. Employees can be frustrated, however, if the leadership style of their superior is inconsistent with the inner expectations of the employees (for example, these employees may see inequities, such as those outside the circle relying on relationships to assign tasks, or managers deliberately hindering employees from completing tasks, or differential leadership giving unfair compensation and promotion opportunities). Especially, when such emotions are not addressed, employees will show a bad work attitude, in part as a way to release their feelings of unbalanced effort and reward. When this situation persists, it becomes a burden to the organization; the overall atmosphere becomes discordant, management instructions cannot be implemented, and grassroots advice cannot reach the top, which can result in the company’s poor operation and failure. In such a situation, how can this management drawback be solved, or how can leaders adopt a more positive incentive strategy?

Data-driven artificial intelligence tools are becoming increasingly more powerful. With stronger capabilities, the utilization of AI technology is expected to boost the world economy (Kliestik et al., 2020; Durana et al., 2021; Lazaroiu et al., 2021; Valaskova et al., 2021). It will have a great impact on enterprises’ added value production (Mitan et al., 2021). Facing a new era and a knowledge-based economy, key elements such as knowledge capital, good corporate governance, and superior-subordinate partnership will be essential for a sustainable business operations (Bulathsinhalage and Pathirawasam, 2017; Xu and Liu, 2020; Krulicky and Horak, 2021; Tijani et al., 2021).

In traditional Chinese culture, leaders have wide-ranging decision-making and discourse power, due to the cultural acceptance of a higher power differential (Wang and Guan, 2018). Leaders tends to divide their subordinates into “insiders” and “outsiders” according to their relationships with them, and then to adopt differentiated management. Sexton et al. (2018) note that employees who have a closer relationship with their leaders may enjoy more resources and opportunities in the organization. Leaders are more active in interacting with “people inside the circle,” and most of them entrust more heavy responsibilities to the insiders and take care of them privately. In response, these insiders may feel that they are more valued and have a deeper sense of gratitude, which may enhance their sense of loyalty to the leader and their commitment to work. This not only improves their own in-role performance, but also creates more benefits and value for the organization. Moreover, the closer these insiders are to their leader, the more they may feel the difference between themselves and other members. Leaders are more rigid with “outsiders” and share less information with them (Kang and Cheung, 2010; Persson and Zhuravskaya, 2016). If the leader’s insiders and outsiders disagree on role identification, it will directly affect the quality of their interactions.

Owing to its being a highly professional and specialized service, medical care contains a large degree of emotional exposure, especially in nursing work between patients and practitioners, so it requires workers to make particular efforts to control their emotions. Therefore, this service sector can be viewed as an emotion-intensive industry (Ashforth and Humphrey, 1993; Wang and Li, 2011). In particular, the emotional labor of employees in the nursing sector has increasingly become an issue that organizational management attaches great importance to. For example, workers might have accumulated grievances at work for various reasons, but due to job responsibilities and requirements, they need to control their emotions and continue to “serve with a smile.” In other words, in order to maintain a proper appearance and state of mind in the work environment, one must buildup oneself psychologically, control negative emotions, and give appropriate feedback to the behavior object (Burns et al., 2019).

The goal of this study is to explore whether a differential leadership style is beneficial to employees’ self-motivation and whether organizational differential motivation will affect performance within roles. Some scholars believe that a differential leadership style does not necessarily only bring negative effects, as it is likely to become an incentive management strategy (De Hoogh et al., 2015). This paper assumes that when leaders classify their subordinates according to standards such as ability and dedication, those “outsiders” who possess a proactive personality may generate some sort of insider status and actively approach the resource controllers. Because employees’ work attitudes and behaviors are affected by the employee-leader relationship, employees who can obtain more resources from leaders will show more enthusiasm. Therefore, employees with a proactive personality consciously improve their work ability, actively change their personal communication behavior, and alter their organizational task environment.

This work contributes to the literature on differential leadership and employee in-role performance in several ways. First, concerning its contribution to theoretical development, previous research work on employee behavior, such as “abusive” and “destructive” leadership, examined the negative aspect of employee job performance. However, similar research on how the proactive personality trait affects the behavior of “outsiders” has not been conducted for a Chinese working environment operating under the theory of differential leadership. Thus, this research offers a theoretical extension to a blank area in the current literature.

Second, concerning its contribution to finding practical application in the context of Chinese localization, we have explored the impact of differential leadership on employees. Since role performance depends on the individual’s self-construction (Turban et al., 2017), adding “proactive personality” as a factor generates a differential incentive strategy and stimulates a “fighting spirit” among employees. This research can provide a reference for the service sector, as well as other related industries, to reduce the disadvantages of conventional differential management.

Third, previous studies have shown that differential leadership does affect employees’ negative behavior. However, our research finds that, as long as differential patterns and partiality are rationally utilized, both “insiders” and “outsiders” can be encouraged to search for their own psychological balance when workers feel fairness is upheld (i.e., meeting their psychological expectations), by changing their workplace behavior and thus promoting the healthy development of the enterprise.

Fourth, in addition to introducing fairness perception as a mediator, this paper also adds “emotional labor” and “proactive personality” as moderator variables. From the perspective of employees’ own feelings and proactive changes, we explore how, under the influence of differential leadership, emotional labor and proactive personality restrain negative effects and generate a kind of motivational effect. This finding may provide some new insights for managers.

### Research questions

Salas-Vallina et al. (2021) pointed out that effective management can increase the effectiveness of subordinates for the benefit of the organization. Employees, in response to the style of leadership they receive, can take actions against situational obstacles through their own perceptions and self-management, which is particularly the case for individuals with proactive personality (Bateman and Crant, 1993). Based on the social exchange theory (SET), we first aim to measure the effect of differential leadership style, upon the relationship between employee proactive personality and employee in-role performance We then explore whether the relationship between the two is positive under the influence of other variables, and whether the sense of organizational justice that is influenced by differential leadership can be used as an intermediary variable in this study. In addition, considering the relationship between employees in the inner circle and outside of it, how does differential leadership create different impacts on the in-role performance of employees?

**Research gaps:** Previous research work on differential leadership has mainly focused on whether loyalty and talent are good criteria for classifying leaders (Cheng and Lin, 1998; Ren et al., 2002), as well as on the impact of differential treatment by leaders on subordinate effectiveness (Asrar-ul-Haq and Kuchinke, 2016). Some studies have tried to detect whether organizational leaders have subordinates of their own (Farahnak et al., 2020). These studies generally found positive effects, although a few showed negative effects. Some works also include job performance (Aarons et al., 2017) and organizational commitment (Keskes et al., 2018). The previous literature on employee behavior identifies the primary positive employee behaviors as in-role behavior, socially beneficial behavior, and organizational citizenship behavior; the primary negative employee behaviors are identified as anti-productive behavior, deviant behavior, and abusive leadership. There is very little research work, however, that has actually considered the specific differential leadership associated with Chinese cultural characteristics. In this case, there are proactive personality traits, acting under different situations and individual perceptions that changed the behavior of “outsiders” as its antecedent variables. Therefore, to make the theory more complete, this paper also uses supplementary variables such as organizational justice and emotional labor.

## Literature review and hypotheses

### Social exchange theory

The earliest theory of social exchange was first proposed by Homans (1958). Its main assumption is that “people are rational” profit-seeking actors, who pay attention to the choice and pursuit of personal interests. In the process of interaction, their main concerns are how to measure the relative benefits between different purposes and actions, and how to conduct exchanges for the highest profit and repayment with the lowest possible cost. Agreements reached by people are the basis for maintaining and stabilizing interpersonal relationships, as well as all social organizations. However, since social exchange theory suggests that human behaviors are governed by the incentive to earn rewards and the desire to receive remuneration, all human social activities can be reduced to a kind of exchange. If so, the social relationship formed in social exchange can also be viewed as an exchange relationship.

Social exchange is part of human behavior. The microstructure of society originates from the exchanges that individuals expect from social rewards. The reason why individuals interact with each other is because they desire to get something from their reasonable exchanges (Erdogan and Enders, 2007). In this regard, social exchange is divided into three forms: (1) social exchange of internal rewards (such as fun, social approval, love, gratitude, etc.), in which the exchange actor takes the process of communication itself as the purpose; (2) social exchange of external rewards (such as money, goods, invitations, assistance, obedience, etc.), in which the exchange actor regards the process of interaction as a means to achieve farther goals (external remuneration provides an objective and independent standard for a person to reasonably choose partners, providing an objective and independent standard); and (3) mixed social exchange, in which actors receives both internal and external remuneration.

Different disciplines have influenced the formation of social exchange theory (SET): (1) Anthropology: Exchange and reciprocity are often the basis of social integration, in primitive tribes, kinship and marriage are an alliance system, and therefore, marriage exchange guarantees alliances and social integration;; (2) Economics: concepts such as compensation or punishment, cost and profit originate from economics; (3) Psychology: Psychological principles can not only explain individual behaviors, but also understand social structures and social changes. People are society, and people’s lives are mostly in the process of interaction with others. All in all, the theory of social exchange is a combination of anthropology, economics, and behavioral psychology, and the interaction between people and the world is regarded as a rational behavior of calculating gains and losses. This idea is the same as that which our research has discussed – the inner and outer circles formed by differential leadership in Chinese culture may shape the differences in employee performance.

From the perspective of social exchange theory (SET), proactive personality is described as “a person who is not restricted by the environment, even if he is treated differently, he can make the environment change” (Bateman and Crant, 1993). Such a person can show initiative and positive work performance (Yang and Chau, 2016; Turban et al., 2017), strong learning motivation (Roberts et al., 2018) and innovative performance (Rodrigues and Rebelo, 2019). Newman et al. (2017) demonstrated that employees with strong proactive personalities are more likely to respond positively to leadership. Furthermore, people with a high level of proactive personality may actively engage in forming new initiatives, recognizing different opportunities, and persevering in achieving their goals (Bateman and Crant, 1993). Therefore, people with proactive personalities may challenge the status quo, while people with passive personalities usually maintain the status quo.

### Factors affecting employee in-role performance

Social exchange theory argues that, when individuals conduct social exchanges, they will first judge their relationship with each other according to the “level of distance,” as the basis and principle of communication or resource allocation (Cropanzano and Mitchell, 2005). Previous studies on in-role performance have focused on employees’ own factors and situational factors (such as incentive policies, people-post matching, leadership, corporate social environment, internal working atmosphere, and opportunities in development). Vigoda (2000) defines in-role performance as behaviors related to employees’ formal role requirements, which are basic job responsibilities and tasks specified in the job description; employees’ performance of such behaviors will result in receiving material and spiritual rewards. Due to cultural differences and perceptions of roles, the division of in-role performance also varies across cultures (Blakely et al., 2005). In terms of employees’ own factors, the employees’ personal characteristics, cognition, attitude, and emotional experience can all be used as influencing factors of their in-role performance. Mindfulness has a positive impact on employees’ in-role performance (Rodrigues and Rebelo, 2019; Jahanzeb et al., 2020); emotional instability will have a negative impact (Judge and Zapata, 2015; Probst et al., 2017); employee personality factors also interact with work stress, which in turn affects performance (Judge and Zapata, 2015). That is to say, competent employees see challenges as an opportunity, and this opportunity is likely to promote personal career development. In order to improve their in-role performance, employees will put in effort and complete tasks to a high quality, which will have a positive impact and their in-role performance will thus be raised. Feng et al. (2019) has shown that, with the increase of challenging stressors, the in-role performance of employees will show an inverted “U” change.

### The impact of proactive personality on employees’ in-role performance

Crant (2000) proposed that proactive behavior is actively transcending the current environment or create a new environment, with strong individual autonomy and purpose, as well as keen insight and an ability to seize opportunities. This paper explores the relationship with in-role performance from three perspectives: personal traits, behavioral perspectives, and action processes of proactive employees. People with proactive personalities have a higher ability to judge the situation. Because of an unwillingness to be restrained, in order to achieve their ideal state or get closer to their goals, this type of worker will actively improve the environment when the organizational situation hinders their interpersonal relationships and career development (Bateman and Crant, 1993). McCormick et al. also confirmed that “proactive personality” has a positive predictive task performance and organizational fairness in individuals with high Situational Judgment Effectiveness (SJE). On the other hand, if “proactive personality” is low, those individuals will not improve their environment or will be assimilated by that environment. The reason is that ability and flexibility are preconditions that determine behavior.

From the perspective of behavior and personal characteristics, employees with proactive personalities will hope to gain status in the organization, and will tend to be valued by leaders. In order to achieve their goals or obtain target resources, they will devote themselves to their work, match themselves to the organizational goals, and be responsible for the results of their labor. Therefore, individuals with proactive personality will change their behavior according to their environment and leadership relationships, will actively seek feedback, and will conduct self-management. They will also formulate plans and implement and anticipate future results. Propelled by high desire, their initiative will become stronger, and they will try to achieve their goals, thereby improving their personal effectiveness and promoting their in-role performance (Crant, 2000). In addition, from the perspective of action processes, the action-goal taken by employees with proactive personality traits is a dynamic process that is planned and expected to produce results, and is future-oriented. For example, when employees find opportunities in organizational activities, they will try to identify the opportunities and the experience needed to complete their task goals and will spend the time and effort needed to achieve their goals and tasks to a high standard and with creativity (Vermooten et al., 2019). In the context of team orientation, employees with proactive personalities are more driven (Turban et al., 2017). However, if employees with proactive personalities do not receive benefits from the organization despite their initiative, it may be due to a mismatch between their personal goals and their organizations’ goals. Therefore, this paper proposes the following hypothesis:

*H1*: Proactive personality has a positive effect on in-role performance.

### The relationship among emotional labor, employee proactive personality, and in-role performance

In service positions in the medical sector, emotional labor is one of the most important work requirements (Hayyat et al., 2017). Morris and Feldman (1996) have shown that emotional labor is influenced by individual traits, situational factors, and sociocultural influences. They emphasized that emotional labor is influenced by the social environment and is a dynamic process. Three factors may affect emotional labor: (1) individual factors, such as gender, age, and personality traits; (2) organizational factors, such as organizational climate and work autonomy; and (3) situational factors, such as emotional events and communicative expectations (frequency, attitude, persistence, etc.). Delgado et al. (2017) believed that emotional labor research in the field of nursing is of great significance. Research shows that women are more likely to express their emotions, and they are more proficient than men in both shallow and deep emotional performances; men are more restrained in regulating their emotions and tend to use shallow acting (Yin et al., 2017; Yang et al., 2018). Diefendorff et al. (2006) and Chapman and Goldberg (2017) also found that emotional labor and personal traits were negative predictors of extraversion, conscientiousness, agreeableness, self-monitoring and superficial acting, while neuroticism positively predicts shallow acting. Employees will interact with the person being served with disguised and false emotions, while extraversion and agreeableness are positive predictors of real emotional performance. Lu and Sun (2021) also confirmed that high agreeableness and conscientiousness in nurses’ personality traits are negatively correlated with surface acting, but positively correlated with deep acting. Therefore, it also shows that the higher the level of emotional intelligence of employees, the higher the deep behavior, and that the less the shallow behavior, the higher the subjective well-being.

Regarding the influence of emotional labor, many scholars believe that it is bidirectional. Erez and Isen (2002) confirmed that emotions may affect cognitive processes, and positive emotions have more persistence and more explicit motivation than neutral emotions. Therefore, it is believed that positive emotions have an impact on goal commitment. Emotional labor occurs when someone’s personal state and work situation are inconsistent; employee emotional instability will have a significant negative impact on in-role performance (Raja and Johns, 2010; Probst et al., 2017). Therefore, the internal response based on environmental stimuli is particularly important, because it can directly affect the display of interpersonal behavior (emotional performance) (Schreuder et al., 2016). However, the effects of superficial acting and deep acting on displaying emotional regulation strategies that meet the situational requirements also differ between individuals (Miller and Gkonou, 2018). Previous studies have found that employees will interact with the people they serve with disguised and false emotions. A real emotional performance of extroversion and agreeableness is a positive predictor, and extraversion is more sensitive to positive emotions. Miller and Gkonou (2018) pointed out the benefit of showing true feelings to clients and showing emotions with true thoughts, noting that extraversion is less painful to positive display rules in the moderating effect of emotional strategies. In addition, studies have found that proactive personality traits are significantly positively correlated with extraversion personality traits (Bateman and Crant, 1993). Exhibitors will also persevere in completing their goals. Thus, it is possible for them to exceed the in-role performance required by their basic job requirements. Employees with strong proactive personalities tend to actively explore or improve the expression of emotional states required by situational constraints at work, so as to promote their expressed emotions to adapt to their service interactions. This trait will encourage employees to internalize the needs and corporate values of the service object and show real emotional experience and empathy, rather than “masking.” Therefore, their emotional labor behavior may have positive utility (Grandey, 2003; Goldberg and Grandey, 2007; Wen et al., 2019). Other studies have found that employees with autonomy have a negative relationship with shallow acting (Goldberg and Grandey, 2007; Muthukrishnan et al., 2018). This can also positively predict deep acting and job satisfaction. Deep acting is further beneficial for avoiding burnout and promoting performance growth within individual roles (Phuoc et al., 2022). This may be because it enables employees to match their real emotions with organizational rules, by learning the internal psychological process of cognition and thinking about phenomena, so as to achieve a unified and coordinated subjective perceptive activity of regulating internal and external emotions. Therefore, this study proposes the following hypotheses:

*H2a*: Superficial acting has a negative moderating effect on proactive personality and employees’ in-role performance.

*H2b*: Deep acting has a positive moderating effect on proactive personality and employees’ in-role performance.

### Differential leadership categorizes subordinates (defines insiders and outsiders)

Jiang and Cheng (2014) pointed out that “insiders” will obtain team resources, but also need to make certain contributions in exchange, while outsiders will have less resource allocation, less strictness, less empowerment, alienation and indifference in interaction, and will be treated in accordance with rules and procedures. Subordinates with closeness, loyalty, and talent may not only positively promote the performance of the supervisor, but also receive generous rewards (Cheng et al., 2002; Epitropaki et al., 2016). For subordinates who only have kinship and loyalty but lack talent, leaders will give more care than to outsiders, based on their personal feelings. Talented people, even if they do not have kinship and loyalty, however, will also be identified and brought into the insider group (Ding and Jie, 2021). The subordinates of the insiders who are cared for privately will show more returns to the leaders when they are favored. When other members observe this phenomenon, the status of being inside or outside the circle becomes a motivator for their actions. Some “outsiders” will take the initiative to approach employees in the circle for their own interests, while continuing to discover interaction rules among people in the circle, which will lead to a good relationship with them, and thus indirectly obtain needed information and resources (Luo et al., 2016).

### The impact of differential leadership on employees’ in-role performance

In Chinese culture, subordinates admire the power of their superiors, which is considered reasonable and an invisible norm (Xiong Chen and Aryee, 2007; Piansoongnern, 2016). Superiors’ treatment of their employees due to the distance of personal relationships is generally accepted by subordinate employees as biased treatment. Also, in an exchange relationship, the subordinates get benefits, and they will repay their superiors through their personal work performance, so as to achieve a reasonable exchange. Valuable communication and positive feedback can strengthen the individual’s sense of competence and autonomy and help to enhance their internal motivation (Matschke and Fehr, 2015).

When superiors treat employees favorably, employees will have a sense of belonging such as being valued, recognized, and understood. They will have a greater right to speak in the organization. They may also participate in leadership decision-making and give more resource allocation to information and material rewards (leadership investment). At the same time, due to the close relationship, they will take the initiative to view themselves as the leader’s “insiders,” and the return to the leader is loyalty, dedication, and better performance (Luo et al., 2016). If an “insider” makes mistakes at work, the mistakes made by the subordinate are less likely to be investigated, and they may even be intentionally overlooked. This custom is considered to be “protecting the calf”; it is tolerant and will help subordinates to find solutions to problems. The subordinates of the “guarded insiders” (those accepting the leader’s reward) will also feel gratitude and recognize their superior’s status. Their trust and respect for the leader will be deepened, and they become more active and involved at work. For outsiders, however, their relationship with the leader is more estranged. Being treated badly, “outsiders” have no first-hand information related to work tasks. Nor are opportunities made available in a timely manner. Since they cannot get resources and rewards in the same way as “insiders,” psychological imbalances may develop, which will affect their work performance and even cause negative behaviors over time (Wang et al., 2021). Matschke and Fehr (2015) found that restrictions, directives, and threats reduce individuals’ subjective motivation and weaken their internal motivation. When employees face a lack of promotion opportunities, their output in the organization will reduce, affecting their in-role performance, due to their inability to develop their own insider identity. Therefore, this paper proposes the following hypotheses:

*H3a*: Differential leadership’s preference (DLP) to insiders has a positive impact on employees’ in-role performance.

*H3b*: Differential leadership’s bias (DLB) against outsiders has a negative impact on employees’ in-role performance.

### Differential leadership and the sense of organizational justice

Adams (1965) defined organizational justice as the perception of fairness in resource distribution and presented the fairest distribution as rewarding people according to their contributions. The more one contributes, the more one should get in return. Cropanzan et al. called employees’ judgments, perceptions, and feelings of fairness in organizations the “fairness perceptions of justice.” Our paper adopts this definition of organizational justice from Cropanzan et al.

Differential leadership usually treats subordinates differently based on characteristics such as their efficiency and similarities to the leaders. This differential management style both is motivated by the purpose of getting the job done and is influenced by purely personal preference. Most of the people inside their “relationship circle” are entrusted with important responsibilities, as well as being fostered and cultivated as cronies who enjoy resources and respect. On the other hand, people outside the circle are treated normally, act according to the rules, and experience a management method that is strict and rigid, or even unreasonable (Jiang and Cheng, 2014; Donia et al., 2016). Therefore, the rewards in the workplace are differentiated. Differential management may increase the phenomenon of members being marginalized within the organization. If people outside the circle find that they are not getting the attention of the leader, they will start to compare their own treatment and information channels to those obtained by the “insiders.” Finding themselves unable to get close to the controller of resources, they can feel lost and can conclude that they are treated unfairly. Some studies have shown that differential leadership results in unfair distribution of material interests and a loss of closeness and trust among “outsiders” (Zheng, 1995; Shu and Lazatkhan, 2017). Akram et al. (2021) believe that, in the context of perceived psychological stress, abusive supervision has both direct and indirect negative impacts on employee creativity. However, distributive and procedural justice are found to be able to mitigate abusive supervision’s negative effects on employee creativity. In an atmosphere of “leadership fairness,” subordinates tend to trust an organization’s distribution process and would tend to put the blame on their own actions if they received a low salary. Thus, they will work even harder (Engelbrecht and Samuel, 2019); this tendency is particularly true among employees who are “insiders” of the leadership. This leads to the following hypotheses:

*H4a*: Differential leadership’s preference (DLP) to insiders will positively affect employees’ sense of organizational justice.

*H4b*: Differential leadership’s bias (DLB) against outsiders will negatively affect employees’ sense of organizational justice.

### The relationship between organizational justice and employees’ in-role performance

A fair relationship is one of the most satisfying social relationships in interpersonal communication. A sense of justice is seen as a motivation that can effectively predict one’s organizational behavior. Albalawi et al. (2019) and Akram et al. (2021) show that distributional justice, procedural justice, leadership justice, and information justice have significant positive correlations with task performance, interpersonal promotion and work dedication. The main reason is that “relationships” are the basis for multi-party social exchanges and judgments. Since the behavioral style of superiors directly affects the action orientation of their employees, superior support has more influence on in-role performance. Yean (2016), Pournader et al. (2020), and Al-Omar et al. (2019) believed that a lack of organizational justice will directly endanger employees’ benefit distribution, interpersonal relationships, and work efficiency. Its organizational fairness is related to a psychological contract. As a kind of implicit but active and flexible virtual contract, it can maintain relationships and output efficiency even within highly emotionally challenging organizations. When there is a sense of a lack of fairness, however, people will feel disappointed and will stop believing that efforts and rewards are proportional, resulting in them no longer producing as many thoughts or behaviors that would have been valuable to the organization. Feelings of being treated unfairly and dissatisfaction with the assigned results may also be compensated for in other negative ways (such as theft or sabotage). Therefore, the following hypothesis is made:

*H5*: Organizational justice positively affects in-role performance.

### The relationship between organizational justice, differential leadership and in-role performance

According to social exchange theory and fairness theory, individuals will return the value they have acquired in order to maintain the distribution of benefits of a social interaction. Zhu and Xie (2018) and Nauman et al. (2020) pointed out that, if employees and the organization have sufficient and fair social exchanges, then employees will feel that they receive sufficient attention and support from the organization. Their sense of identity and responsibility will be significantly enhanced and will spontaneously produce positive behaviors that are beneficial to the organization. This kind of fairness will make employees feel that they have the responsibility and obligation to do their work better and to contribute to the development of the organization. In contrast, if the sense of justice within the organization is weak, then employees may feel that they are incapable of changing the atmosphere, resulting in two psychological behaviors. The first of these behaviors is positive: a growing effort to achieve a stronger right to speak and to try to improve unfair environments in the organization, opening a positive path for later employees. The second behavior is negative, and can be divided into two aspects: (1) an attitude which believes that, in an unfair environment, no matter how hard you try, you will not be able to achieve your ambitions and prospects, but that you cannot give up due to social life factors; and (2) anti-productive behavior (Pournader et al., 2020) that can induce a destructive behavior in the organizational environment. This can spread or instigate other employees to also act poorly.

To sum up, from the perspective of social exchange, the determination of fairness depends on whether each person receives equal benefits and distribution during exchange processes. The distribution of benefits is based on contributions, but both parties are restricted by social rules. It also depends on the ownership of the resource and the relationship with the dominant player during the exchange. When employees feel that the interpersonal relationship is unbalanced, their sense of justice will be lacking and their work engagement will decrease, resulting in lower in-role performance. Therefore, this paper proposes the following hypotheses:

*H6a*: Organizational justice has a mediating effect on the relationship between differential leadership’s preference (DLP) for insiders and employees’ in-role performance.

*H6b*: Organizational justice has a mediating effect on the relationship between differential leadership’s bias (DLB) against outsiders and employees’ in-role performance.

### The moderating effect of employees’ proactive personality

Employees with proactive personalities have inherent positive tendencies and the characteristic of creating a favorable environment, so their ability to adjust to the environment is also high. The differential treatment of “insiders” and “outsiders” by differential leadership has also become a means of differential incentive management. Because of the existence of favorable treatment toward “insiders,” when faced with differential treatment by differential leaders, employees with high proactive personality are more likely to take the initiative (Li et al., 2018; Wang and Lei, 2021). Therefore, if people outside the circle also have a strong proactive personality, they will use growth needs as the motivation to change or win the favor of the leader and change the leader’s classification of themselves. In this context, the sense of organizational justice is considered to be a very important situational factor that affects the expression of individual characteristics. Employees with high proactive personality will find ways to meet and achieve their expected goals, and when they perform, they will also evaluate and segment the expected target to offset the initial high sense of unfairness and to gradually reduce or replace it. Also, because they are unwilling to be restrained, when the organization hinders their interpersonal relationships and career development, they will actively improve the environment. They hope to gain achievements and status in the organization, to be valued and promoted by leaders, and to achieve their goals or obtain target resources. At the same time, employees who are “insiders” with proactive personalities may, based on their own ability, take their favorable treatment from their leader for granted and view such favoritism as fairness, even when they have obtained more resources than others. Individuals with a lower proactive personality exhibit the opposite behavioral pattern. They fail to recognize opportunities, appear relatively passive, and like to rely on others to drive change. Therefore, this paper proposes the following hypotheses:

*H7a*: Proactive personality positively moderates the relationship between differential leadership’s preference (DLP) to insiders and organizational justice.

*H7b*: Proactive personality positively moderates the relationship between differential leadership’s bias (DLB) against outsiders and organizational justice.

## Methodology

In this study, we used PROCESS SPSS MACRO for analysis. All dimensions were averaged by the method of ITEM PARCELING, and we turned this into an observation variable for analysis. Therefore, the graphics are rendered in squares that are for path analysis.

### Sampling and data collection

The data used in this study were collected from supervisors and subordinates of well-known hospitals (one of the research assistants was previously a nurse) and of health institutions in Beijing in China. A total of 900 questionnaires for supervisors and subordinates were distributed (250 for supervisors and 900 for subordinates). After deducting invalid questionnaires, a total of 524 valid samples (192 questionnaires for supervisors and 524 questionnaires for subordinates) were obtained; the recovery rate of valid samples was 58.2%. The subjects of this research questionnaire were the paired questionnaires of direct supervisors and subordinates. A set of valid questionnaires was obtained when a supervisor matched 3–5 subordinates (if the supervisor did not answer or if the number of subordinates who answered was insufficient, then the group of data was deleted). In addition to collecting data from different sources, this study also collected data at different time points (that is, at a 1-month interval), so as to avoid the problem of common method variation. The procedure for distributing the questionnaires for this study was as follows:

1. This study first contacted the liaisons of hospitals and health institutions in Beijing, and screened the eligible subjects for the questionnaire. The subject must be one supervisor with at least 3 subordinates. After asking for the number of supervisors and subordinates who were interested in participating in this study, the questionnaires were sent to the liaisons of each institution, for assistance in forwarding the questionnaires to the study participants.
2. Each questionnaire included the purpose of the research and the method for answering the questionnaire. In the first stage, after all subjects had filled in their basic information, the supervisors conducted a self-assessment on differential leadership, while the subordinates answered an assessment on their sense of organizational justice. After filling out all the questions, each participant put the questionnaire in an envelope and returned it to the contact person. In the second stage, 1 month after completion of the questionnaire, the subordinates would evaluate their in-role performance, proactive personality, and emotional service, then return the questionnaire to the contact person in an envelope.

### Data analysis

Stratified random sampling was used for this study. The samples came from six main urban areas in Beijing (Dongcheng, Xicheng, Chaoyang, Fengtai, Shijingshan, and Haidian Districts), and the hospitals in the 6 urban areas were classified according to their grade levels (Grade 1 and below, Grade 2, and Grade 3 and above). According to the Information Center of the Beijing Municipal Health Commission, there were about 297,000 registered health technicians in Beijing in 2020. Among them, there were 189,000 people in the six major urban areas, with the grade levels of first-level-and-below, second-level, and third-level-and-above accounting for about 10, 35, and 55%, respectively. Ghiselli et al. (1981) proposed that the number of questionnaires to be distributed should be at least 5–10 times the number of itemized questionnaires. This study originally had a total of 58 topics, so 900 questionnaires were randomly distributed. The first phase began distribution in early January 2021, breaking up the questions and distributing 300 copies at random. In order to recover real and effective data smoothly, each participant was offered a “red packet” (a Chinese custom of showing appreciation) as a reward after completing and returning a questionnaire. By February 6, 250 questionnaires had been received. After eliminating unqualified questionnaires, such as those with confusing basic information or that had been filled-in incompletely or left blank, we finally recovered 213 valid questionnaires; so, the effective questionnaire rate was 71%. For the second stage, after a 1-month interval, the questions were broken up and 600 copies were distributed randomly on February 10. By March 30, 477 copies were recovered. After excluding invalid questionnaires with missing values, wrong answers or random answers, we recovered a total of 311 valid questionnaires; the effective rate of the questionnaire was 51.83%.

The total number of participants in this study was 524. There were 167 males and 357 females, and their majors varied. The 524 participants were distributed thusly by age: (below 20 years old: 59, 11.3%; 21–30: 313, 59.7%; 31–40: 112, 21.4%). Most respondents were undergraduates (252, 48.1%). In seniority, 401 had served 2 to 10 years (76.6%). Regarding position, “without any title” were 325 (62%); 134 were at a basic level (25.6%). In terms of hospital scale, most of the participants were from Grade 2 and above (473, 90.3%).

### Instrument and measurement

#### Dependent variables

The measurement method of the research constructs was developed based on previous reports in the literature. All items were assessed based on a five-point Likert scale ranging from “strongly disagree” (1) to “strongly agree” (5). To assure the consistency of the measure used, translation and back-translation procedures between the Chinese and the English language (Van de Vijver and Leung, 1997) were applied. Furthermore, three business administration professors helped to revise the descriptions of the scale items to confirm the construct’s validity. Finally, a pretest was done among 130 medical staff. A reliability analysis showed no item with an item-to-total value below 0.30. Employee in-role performance was measured with the use of five items adopted from Eisenberger et al. (2010): 1. Completing the assigned tasks successfully. 2. Fulfilling the job content specified by the job responsibilities. 3. Meeting the performance requirements set out in the job. 4. Accomplishing the tasks expected by the organization. 5. Sometimes neglecting some of the responsibilities that should be performed at work occasionally*. The fifth question is given an asterisk (*) because it is a reverse question, used to verify whether the answer-logic of the subjects is consistent, between before and after.

#### Independent variables

**Proactive personality** was measured with the use of 17 items adopted from Bateman and Crant (1993). The scale was adapted to the localization context, and after EFA reduction, 8 items were selected: 1. “No matter what the situation is, as long as I decide things, I will put them into practice.” 2. “I am willing to stand up for my ideas even in the face of opposition.” 3. “I am used to standing up for others when it comes to giving advice and implementing new projects*.” 4. “No matter where I am, I have a strong ability to drive organizational change to happen.” 5. “If I see something unreasonable, I will change it.” 6. “There is nothing more exciting than seeing your ideas come true.” 7. “I am always looking for new ways to improve my work.” 8. “I am able to keenly identify and grasp opportunities for learning and advancement at work.” Note, again, that the * indicates a reverse question.

**Differential leadership** was measured with the use both of 11 items that are biased towards outsiders which have been adopted from Jiang and Cheng (2014) and also of the 10 items that are biased toward insiders which have been adopted from Jiang and Zhang (2010). After context modification, the measurement basis of differential leadership was generated. *Preference to insiders*: 1. Greeting employees and having frequent contact and interaction. 2. Spending more time on personal experience sharing and guidance. 3. Helping and supporting in emergencies. 4. Assigning a subordinate to convey work information frequently. 5. Giving more opportunities to get rewards and promotions. 6. Assigning more important and easy-to-achieve tasks. 7. Giving large rewards. 8. Lessening penalties for work mistakes. 9. Rarely getting blamed for mistakes at work, and 10. Rarely pursuing subordinates for mistakes. *Bias against outsiders:* 11. Having an indifferent attitude and keeping a certain distance*. 12. Being less likely to mentor or share work experience and knowledge skills*. 13. Being less likely to help solve problems at work*. 14. More often withholding key information at work*. 15. In the performance appraisal of subordinates, being less merciful, and handling everything in accordance with the rules and regulations*. 16. When subordinates review their own mistakes, strictly accusing without mercy*. 17. Being less likely to be perceived as needing assistance when things go wrong*. 18. More often ignoring work problems reported by subordinates*. 19. Making frequent public criticism and censure*. 20. Often being sarcastic at work*; and 21. More often arranging complex and difficult tasks that are not easy to complete*. Note: * indicates a reverse question.

**Emotional labor** was measured with the use of 8 items adopted from Grandey (2003). Grandey (2003) divides emotional labor into two dimensions: surface behavior and deep behavior. This division fits the Chinese context. *Shallow play:* 1. “Even if I am in a bad mood, I will show a happy appearance.” 2. “At work, no matter how complicated my inner feelings are, I will play appropriate attitudes and emotions to meet the emotional expression requirements of the organization.” 3. “In order to express the emotions required for work, I wear a ‘mask’.” 4. “Emotions expressed at work do not match my inner feelings.” *Deep play:*5. “When facing colleagues and work, the emotions I express are from the heart.” 6. “When there is a problem at work, I will try my best to overcome the bad emotions and actively solve the problem.” 7. “For the sake of work, even if I am in a bad mood, I will try to adjust my mood.” 8. “When you are in a bad mood, by communicating with colleagues and actively engaging in work, the negative emotions will be reduced.”

**Organizational justice** was measured with the use of 10 items adopted from Niehoff and Moorman (1993) and Wang (2009). The 10 items are as follows: 1. “Leaders show me respect when making a decision about my job.” 2. “When making a decision related to my job, the leader chooses to do so in a practical way.” 3. “Leaders will treat me fairly and equitably when making a decision related to my job.” 4. “Organizational leaders make work decisions in an unbiased state.” 5. “Decisions made by the organization apply consistently to all employees.” 6. “The top leaders of the organization will collect sufficient and correct information before making decisions.” 7. “When subordinates request, the leader will clarify some relevant decision-making supplementary information.” 8. “I think the workload assigned to me is fair.” 9. “I think my salary is fair.” 10. “My work rights and responsibilities are relatively fair.”

#### Common method variation

In order to avoid the validity problems caused by questionnaires coming from the same source or the same test environment, this research uses a reverse questionnaire design and a post-event statistical control to reduce or even avoid the influence of CMV. This step uses the Harman (1976) one-factor test. It can be seen from Table 1 that the explained variance value of the first principal component before rotation is 32.674% < 40%, indicating that the common method bias test was passed.

**Table 1:** Common method bias tests.

Element	Initial eigenvalues	% of variance	Cumulative %	Extract the square	% of variance
1	16.99	32.674	32.674	16.99	32.674
2	3.827	7.361	40.034	3.827	7.361
3	3.516	6.762	46.796	3.516	6.762
4	2.645	5.087	51.884	2.645	5.087
5	2.389	4.593	56.477	2.389	4.593
6	1.745	3.355	59.832	1.745	3.355
7	1.399	2.690	62.522	1.399	2.690

Compiled by this study.

### Data collection

The survey instrument contained 48 items (totaling 7 constructs) adapted from previous studies (see Appendix B). The survey measured participants’ perceptions with a 5-point Likert scale, ranging from totally disagree to totally agree. Higher scores on this instrument indicated more positive perceptions. All data were collected by online survey.

## Results

### Data analysis

**Figure 1:**
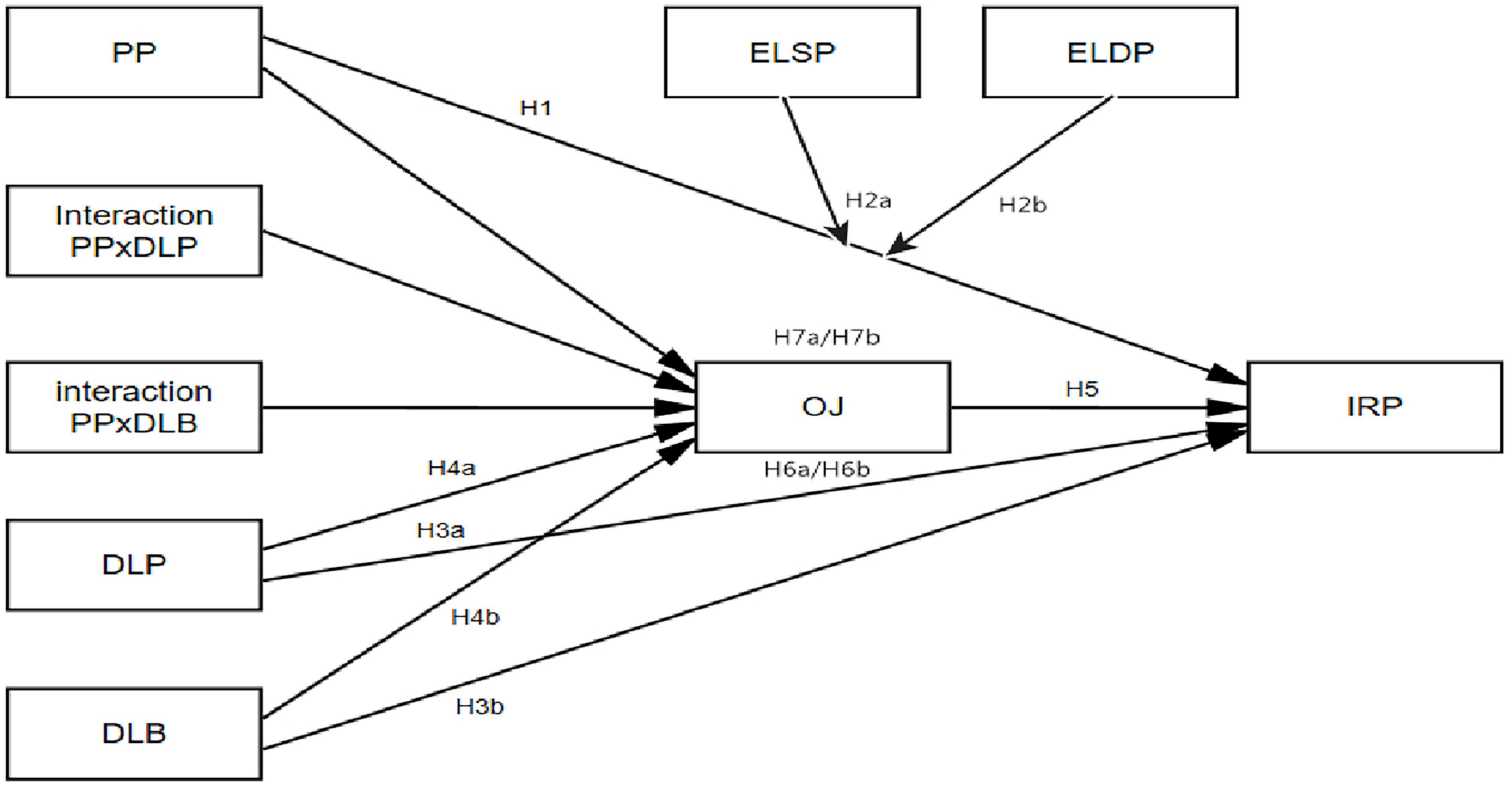
Research framework. Compiled by this study.

This study uses structural equation modeling (SEM) to test the measurement model’s reliability and validity. (Note that SEM is only used for validation; SPSS PROCESS MACRO is used to test mediation and moderation, in keeping with the research framework presented in Figure 1). Amos 24 was used to evaluate the measurement model. In this model, if the chosen indicators for a construct do not measure that construct, then the testing of that structural model will be meaningless (Jöreskong and Sörbom, 1998). Thus, the first-step in the modeling approach, as recommended by Anderson and Gerbing (1988) and McDonald and Ho (2002), was followed by carrying out a confirmatory factor analysis (CFA) to provide an assessment of convergent and discriminant validity, and then the PROCESS was carried out to provide the path coefficients, mediations, and moderations.

### Measurement model

The measurement model was assessed using AMOS 24.0 with maximum likelihood estimation (MLE) in terms of individual item factor loadings, reliability of measures, convergent validity, and discriminant validity. MLE allows computation of assorted indices of goodness-of-fit and the testing of the significance of loadings and correlations between factors, but it requires an assumption of multivariate normality. Table 2 presents a summary of the unstandardized factor loadings, standard error, significance test, standardized factor loadings, composite reliability (CR), and average variance extracted estimates (AVE). CR reflects the internal consistency reliability among indicators of a construct. As shown in Table 2, all values of the CR exceed 0.7, thus showing good reliability for all six constructs. Fornell and Larcker (1981) proposed three measures for assessing convergent validity of the measurement items: (a) item reliability of each measure, (b) composite reliability of each construct, and (c) the average variance extracted (AVE). On the reliability of the items, the standardized loading values exceeded 0.6 that are ranging from 0.672 to 0.840, the recommended threshold by Gefen et al. (2000), thus demonstrating convergent validity at the item level. For composite reliability, all values exceeded 0.7 that are ranging from 0.804 to 0.924, the recommended threshold by Nunnally and Bernstein (1994). Lastly, on the average variance extracted, all values exceeded 0.5, ranging from 0.539 to 0.639. Given the satisfaction of three criteria, the convergent validity for the proposed constructs of the measurement appears to be adequate.

**Table 2:** Reliability and convergent validity.

Construct	Item	Significance test of parameter estimation				Item reliability		Composite reliability	Convergence validity
		Unstd.	S.E.	Unstd./S.E.	*p*	STD.	SMC	CR	AVE
DLB	DLB1	1.000				0.691	0.477	0.928	0.539
	DLB2	0.967	0.066	14.727	0.000	0.676	0.457		
	DLB3	1.271	0.079	16.069	0.000	0.754	0.569		
	DLB4	1.271	0.080	15.973	0.000	0.749	0.561		
	DLB5	1.085	0.071	15.265	0.000	0.710	0.504		
	DLB6	1.114	0.074	14.998	0.000	0.699	0.489		
	DLB7	1.173	0.074	15.941	0.000	0.747	0.558		
	DLB8	1.210	0.077	15.627	0.000	0.727	0.529		
	DLB9	1.289	0.079	16.275	0.000	0.764	0.584		
	DLB10	1.231	0.072	17.008	0.000	0.791	0.626		
	DLB11	1.217	0.075	16.206	0.000	0.758	0.575		
DLP	DLP1	1.000				0.780	0.608	0.924	0.550
	DLP2	0.948	0.052	18.395	0.000	0.748	0.560		
	DLP3	0.945	0.052	18.145	0.000	0.743	0.552		
	DLP4	0.917	0.052	17.567	0.000	0.729	0.531		
	DLP5	0.872	0.052	16.930	0.000	0.708	0.501		
	DLP6	0.949	0.053	17.947	0.000	0.738	0.545		
	DLP7	0.962	0.052	18.512	0.000	0.760	0.578		
	DLP8	0.814	0.050	16.359	0.000	0.683	0.466		
	DLP9	0.871	0.050	17.377	0.000	0.720	0.518		
	DLP10	0.981	0.049	20.083	0.000	0.800	0.640		
ELDP	ELDP1	1.000				0.839	0.704	0.876	0.639
	ELDP2	0.842	0.043	19.657	0.000	0.764	0.584		
	ELDP3	0.810	0.041	19.574	0.000	0.772	0.596		
	ELDP4	0.947	0.045	21.060	0.000	0.820	0.672		
ELSP	ELSP1	1.000				0.780	0.608	0.804	0.507
	ELSP2	0.832	0.060	13.909	0.000	0.672	0.452		
	ELSP3	0.811	0.057	14.158	0.000	0.678	0.460		
	ELSP4	0.874	0.059	14.888	0.000	0.714	0.510		
IRP	IRP1	1.000				0.764	0.584	0.873	0.580
	IRP2	0.869	0.053	16.556	0.000	0.730	0.533		
	IRP3	0.867	0.052	16.660	0.000	0.723	0.523		
	IRP4	0.963	0.055	17.648	0.000	0.764	0.584		
	IRP5	0.988	0.052	19.033	0.000	0.823	0.677		
OJ	OJ1	1.000				0.817	0.667	0.922	0.543
	OJ2	0.808	0.046	17.577	0.000	0.700	0.490		
	OJ3	0.767	0.044	17.339	0.000	0.692	0.479		
	OJ4	0.921	0.046	19.905	0.000	0.767	0.588		
	OJ5	0.854	0.046	18.453	0.000	0.726	0.527		
	OJ6	0.812	0.044	18.465	0.000	0.723	0.523		
	OJ7	0.804	0.044	18.070	0.000	0.714	0.510		
	OJ8	0.814	0.044	18.330	0.000	0.722	0.521		
	OJ9	0.844	0.046	18.241	0.000	0.720	0.518		
	OJ10	0.900	0.044	20.427	0.000	0.781	0.610		
PP	PP1	1.000				0.837	0.701	0.922	0.596
	PP2	0.943	0.046	20.456	0.000	0.768	0.590		
	PP3	0.847	0.045	18.878	0.000	0.727	0.529		
	PP4	0.829	0.044	18.650	0.000	0.718	0.516		
	PP5	0.866	0.043	19.984	0.000	0.761	0.579		
	PP6	0.906	0.046	19.686	0.000	0.747	0.558		
	PP7	0.983	0.048	20.645	0.000	0.771	0.594		
	PP8	0.984	0.041	23.786	0.000	0.840	0.706		

Unstd, Unstandardized factor loadings; Std, Standardized factor loadings; SMC, Square Multiple Correlations (square of STD); CR, Composite Reliability; AVE, Average Variance Extracted.

For the discriminant validity, the square root of the AVE for a given construct was compared with the correlations between the construct and other constructs (Fornell and Larcker, 1981). If the square root of the AVE of a construct is greater than the off-diagonal elements in the corresponding rows and columns, then this indicates that that construct is more-closely related with its indicators than with the other constructs. In Table 3, the diagonal elements in the matrix are the square roots of the AVE. Because the square roots of the AVE are higher than the values of its corresponding rows and columns, discriminant validity is found to be satisfactory for all constructs.

**Table 3:** Discriminant validity of Fornell and Larcker criteria.

Construct	Pearson product correlation coefficients						
	PP	DLP	DLB	OJ	ELSP	ELDP	IRP
PP	**0.772**						
DLP	0.485	**0.734**					
DLB	−0.510	−0.502	**0.742**				
OJ	0.438	0.540	−0.626	**0.737**			
ELSP	0.283	0.230	−0.366	0.267	**0.712**		
ELDP	0.490	0.345	−0.444	0.308	0.494	**0.799**	
IRP	0.518	0.442	−0.624	0.527	0.412	0.588	**0.762**

The diagonal elements are the square root of AVE, the off-diagonals are Pearson coefficients Structural model.

Compiled by this study.

### Direct effect analysis

Before we tested mediation and moderation, the path coefficients of independent variables to dependent variable were computed. The full model is comprised of two sub-models. Model 1 entails the regressing of the OJ onto PP, PP × DLP, PP × DLB, DLP and DLB. Model 2 involves the regressing of Y onto PP, OJ, DLP, and DLB. The obtained results are presented in Table 4.

**Table 4:** Regression coefficients.

DV	IV	Coeff	SE	*t*	*p*	LLCI	ULCI	*R* ^2^
OJ	Constant	2.960	0.236	12.517	0.000	2.495	3.424	0.455
	PP	0.102	0.033	3.050	0.002	0.036	0.168	
	**PP**×**DLP**	**0.199**	**0.036**	**5.572**	**0.000**	**0.129**	**0.270**	
	**PP** × **DLB**	**0.187**	**0.031**	**5.980**	**0.000**	**0.125**	**0.248**	
	DLP	0.328	0.041	8.067	0.000	0.248	0.408	
	DLB	−0.311	0.038	−8.084	0.000	−0.386	−0.235	
IRP	Constant	2.948	0.313	9.427	0.000	2.334	3.563	0.393
	PP	0.195	0.039	5.072	0.000	0.120	0.271	
	OJ	0.180	0.050	3.593	0.000	0.082	0.279	
	DLP	0.085	0.049	1.753	0.080	−0.010	0.181	
	DLB	−0.361	0.048	−7.604	0.000	−0.455	−0.268	

Compiled by this study.

The coefficient for all independent variables were significant to OJ, including the interaction between PP and DLB, PP and DLP were significant. In the second part regression, IRP was regressed on PP, OJ, DLP and DLB. The coefficients were significant here also, but with DLP➔IRP.

Two interactions to OJ are significant: PP × DLP ➔OJ (*β* = 0.199, SE = 0.036, t = 5.572, *p* < 0.001, [0.129 0.270]) and PP × DLB ➔OJ (β = 0.187, SE = 0.031, *t* = 5.980, *p* < 0.001, [0.125 0.248]).

### Mediation and moderation analysis

In the mediating and moderating analysis, we examined the mediation effect first. Mediation analysis is used to identify and explicate the relationship between the dependent variable Y and an independent variable X, which may be affected *via* the interaction of a third variable M. Here, M is a mediating variable, and it represents a mechanism through which X affects Y. In our current study, “PP,”"DLP,”"DLB” are independent variables and “PP × DLP” and “PP × DLB” are our interactions impacts from “OJ”; with “OJ” acting as a mediator variable, which further affects the “IRP.”

We conducted path analysis by PROCESS 3.5 macro (Hayes, 2012) to test these indirect effects, and we determined statistical significance by bootstrapping using 5,000 resamples (Hayes, 2009). In PROCESS v3.5 built-in models, we could find no model that fit to our hypothesis research model. Thus, we used PROCESS syntax to do the work; that syntax is presented in Appendix A.

### Indirect effect analysis

We used 5,000 times bootstrap samples in the present study and determined the mediating effect of the 95% confidence interval. The results are shown in Table 5. The first column is the direct and indirect effects, the second through fourth columns are, respectively, the point estimate, standardize error, and confidence intervals. If confidence intervals do not include 0, then this means that an indirect effect is supported. The last 3 columns report the Sobel z tests; if z > 1.96 and *p* < 0.05, then an indirect effect is supported. Our five hypotheses were all supported by the results, as Table 5 shows. From Table 5, it can be seen that the indirect effect of the mediation path “Preference for Insiders-Organizational Fairness-In-Role Performance” is 0.059, at a 95% confidence interval. The upper interval is 0.104 and the lower interval is 0.022; thus it does not include any 0 values. Moreover, the *p* value is less than 0.05, all of which indicates that there is a significant mediating effect. In addition, the direct effect is 0.085. The 95% confidence interval of Bias-correction contains 0, so the direct effect is not significant. Therefore, H6a is verified and is fully intermediary. The indirect effect of the mediation path “preference to outsiders-organizational justice-in-role performance” is −0.056, at a 95% confidence interval; the upper interval is −0.023, and the lower interval is −0.096. It does not include any 0 values and the *p* value is less than 0.05, thus indicating that there is a significant mediation effect. In addition, the direct effect is −0.361; the 95% confidence interval of Bias-correction does not contain a 0, so the direct effect is significant, and the estimated value becomes smaller. Therefore, it is verified that H6b is a partial intermediary.

**Table 5:** Mediating effects.

Path	Bootstrap 5,000 times confidence interval				Sobel *z* test		
	Estimate	BootSE	BootLLCI	BootULCI	SE	*Z*	*p*
*Direct effect*							
PP → IRP	0.195	0.039	0.12	0.271			
DLP → IRP	0.085	0.049	−0.01	0.181			
DLB → IRP	−0.361	0.048	−0.455	−0.268			
*Indirect effect*							
PP→OJ→IRP	**0.018**	**0.010**	**0.002**	**0.040**	**0.008**	**2.275**	**0.023**
PP×DLP→OJ→IRP	**0.036**	**0.012**	**0.014**	**0.063**	**0.012**	**2.986**	**0.003**
PP×DLB→OJ→IRP	**0.034**	**0.012**	**0.012**	**0.061**	**0.011**	**3.048**	**0.002**
DLP→OJ→IRP	**0.059**	**0.021**	**0.022**	**0.104**	**0.018**	**3.261**	**0.001**
DLB→OJ→IRP	**−0.056**	**0.019**	**−0.096**	**−0.023**	**0.017**	**−3.262**	**0.001**

Compiled by this study.

### Moderating effect

In order to better display the moderation effect, we followed Aiken et al. (1991) procedures and examined at one standard deviation (SD) above the mean, at the mean, and at one SD below the mean, for the personality values used as the moderator variable of interest. This analysis was to determine if the slopes of the regression equations for high and low values of the interaction differed from zero. The present study had explores two potentially moderation effects, PP × ELSP➔IRP and PP × ELDP➔IRP, and the analysis results are presented in Table 6 below (Other potential moderating effects could also be analyzed in future works). PP **×** ELSP➔IRP (*β* = −0.154, SE = 0.035, *t* = −4.394, *p* < 0.001, [−0.223–0.085]) and PP **×** ELDP➔IRP (*β* = 0.112, SE = 0.034, *t* = 3.327, *p* = 0.001, [0.046 0.178]), *p* < 0.05 and the bias-correction results do not include 0, which indicates that the moderating effect exists.

**Table 6:** Moderating effect.

DV	IV	Coeff	SE	*T*	*p*	LLCI	ULCI	*R* ^2^
OJ	Constant	2.960	0.237	12.517	0.000	2.495	3.424	0.455
	PP	0.102	0.034	3.050	0.002	0.036	0.168	
	PP **×** DLP	0.199	0.036	5.572	0.000	0.129	0.270	
	PP **×** DLB	0.187	0.031	5.980	0.000	0.125	0.248	
	DLP	0.328	0.041	8.067	0.000	0.248	0.408	
	DLB	−0.311	0.039	−8.084	0.000	−0.387	−0.235	
IRP	constant	1.303	0.478	2.727	0.007	0.364	2.242	0.488
	PP	0.221	0.120	1.835	0.067	−0.016	0.457	
	OJ	0.177	0.047	3.785	0.000	0.085	0.268	
	ELSP	0.647	0.135	4.798	0.000	0.382	0.912	
	**PP×ELSP**	**−0.154**	**0.035**	**−4.394**	**0.000**	**−0.223**	**−0.085**	
	ELDP	−0.136	0.123	−1.112	0.267	−0.377	0.104	
	**PP×ELDP**	**0.112**	**0.034**	**3.327**	**0.001**	**0.046**	**0.178**	
	DLP	0.072	0.045	1.588	0.113	−0.017	0.161	
	DLB	−0.238	0.046	−5.182	0.000	−0.328	−0.148	

Compiled by this study.

### Analysis of research results

From the results of the path analysis in Table 4, we see that proactive personality has a significant positive impact on employee performance within roles. Its standardized path parameter β value is 0.195, SE = 0.039, t = 5.072, *p* < 0.001, which means that H1 is verified. We also see from the path analysis results in Table 6 that PP **×** ELSP➔IRP (β = −0.154, SE = 0.035, t = −4.394, p < 0.001, [−0.223–0.085]. This shows that the interaction item “proactive personality” **×**” superficial acting” has a significant negative effect on in-role performance. Therefore, the moderator variable “shallow acting” has a significant negative moderating effect on the relationship between “proactive personality” and “in-role performance.” Hypothesis H2a is thus supported. PP **×** ELDP➔IRP (*β* = 0.112, SE = 0.034, *t* = 3.327, *p* = 0.001, [0.046 0.178], *p* < 0.05 and the 95% confidence result does not include 0, so the moderated effect exists. It shows that the interaction item “proactive personality” **×** “deep-play” has a significant effect on in-role performance. Hence, Hypothesis H2b is also supported.

Also from the results of the path analysis in Table 4, we see that the standardized path coefficients of differential leadership’s preference to insiders and employees’ in-role performance are *β* = 0.085, SE = 0.049, *t* = 1.753, *p* < 0.08, 0.099, *p* value is 0.04 < 0.05, [−0.010 0.181], which means that Hypothesis H3a is invalid. Perhaps when the subordinates feel that the leader has a preference for them or that they have a special relationship with their leader, then they may become arrogant, thinking that they can survive in the business without working hard. In that case, then, their performance would be worse than others, over time. Real life anecdotal experience suggests that such people do exist.

There is a significant negative relationship between differential leadership’s biases against outsiders and employees’ in-role performance. From Table 4, we see that its standardized path coefficient *β* = −0.361, SE = 0.048, *t* = −7.604, *p* < 0.001, [−0.455–0.268], which means that Hypothesis H3b is supported. When employees are treated badly, they will feel insecure, uncertain, anxious, and other negative emotions, which will then affect their role behaviors and even produce negative behaviors (Zheng, 1995; Jiang and Cheng, 2014). From Table 4, we see that the biased treatment of differential leaders will significantly and positively affect employees’ sense of organizational justice. Its normalized path coefficient *β* = 0.328, SE = 0.041, *t* = 8.067, *p* < 0.001, [0.248 0.408], which means that Hypothesis H4a is valid. When employees are viewed as outsiders, differential leadership treats outsiders in a negative way, which will negatively affect employees’ sense of organizational justice. Its normalized path coefficient *β* = −0.311, SE = 0.038, *t* = −8.084, *p* < 0.001, [−0.386–0.235], which means that Hypothesis H4b is also valid. When outsiders find a large gap (in terms of rewards and status they have received) between themselves and the insiders, they will feel unfairly treated. This feeling directly affects the actions and attitudes of outsiders. We can also see from Table 4 that *β* = 0.180, SE = 0.050, *t* = 3.593, *p* < 0.001, [0.082 0.79], indicating that the sense of organizational justice has a significant positive impact on the employees’ in-role performance, so hypothesis H5 is also established.

From Table 6, the interaction terms between independent variables and moderator variables, the standardized path coefficients of proactive personality **×** differential leader’s preference for oneself and organizational justice sense are *β* = 0.199, SE = 0.036, *t* = 5.572, *p* < 0.001, [0.129 0.270], which shows that Hypothesis H7a is valid. We can also see from Table 6 that the interaction term of the independent variable and the moderator variable, the standardized path coefficient of proactive personality **×** differential leadership to outsiders’ bias towards organizational justice are *β* = 0.187, SE = 0.031, *t* = 5.980, *p* < 0.001, [0.125 0.248], verifying that hypothesis H7b is also valid.

**Figure 2:**
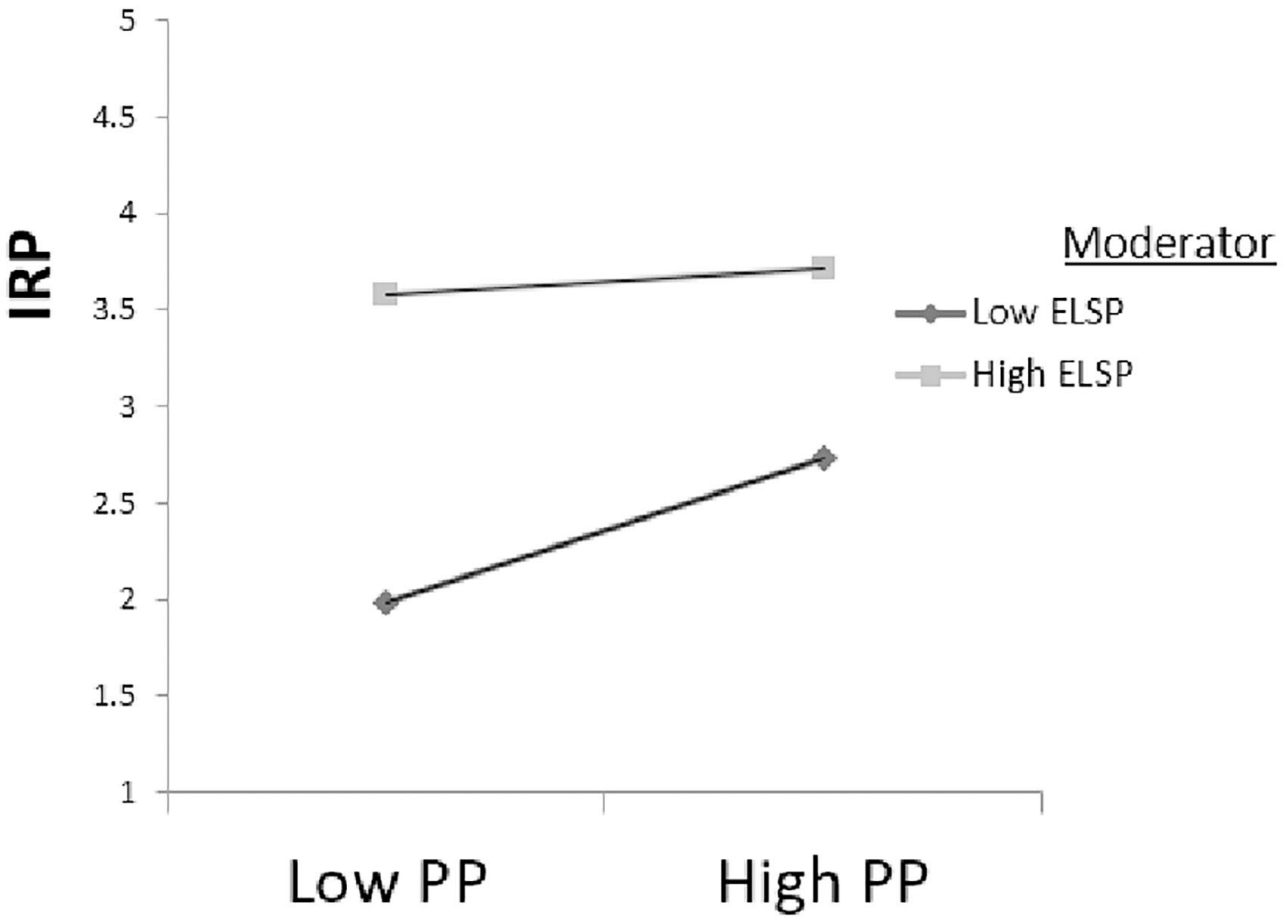
Proactive Personality **×** Superficial Play Interaction Effects. Compiled by this study.

**Figure 3:**
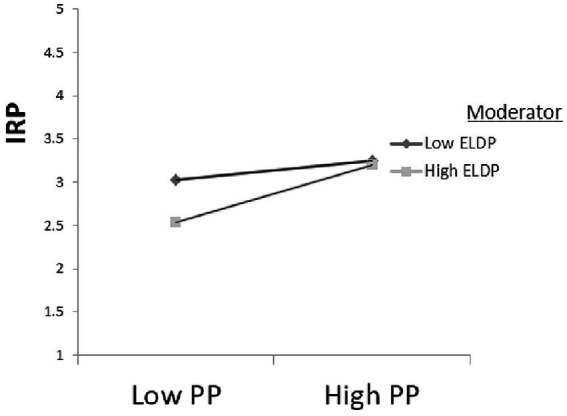
Proactive Personality **×** Deep Play Interaction Effect. Compiled by this study.

In terms of the adjustment effect, Figure 2 shows that, when the level of “proactive personality” is low, low-level “superficial acting” has better in-role performance than “deep-level acting.” For superficial acting in a high degree of “proactive personality,” the in-role performance is better than a low degree of “proactive personality.” Figure 3 shows that low-level superficial acting has higher in-role performance than higher superficial acting at a lower level of “proactive personality.” Nonetheless, for higher “proactive personality,” the performance of the two roles tends to be consistent.

## Discussion and conclusion

The findings reveal that, when faced with difficulties or unfair treatment, employees with proactive personality traits may take the initiative to change situational obstacles, to improve current difficulties and to positively impact their in-role performance. This is consistent with the research proposed by Ghosh et al. (2017), who argue that subordinates may hold key information, and under the influence of certain factors, some negative behaviors mat manifest at work. There are always differences among people with different personalities, so even if the manager treats all of them poorly, not everyone’s work performance will suffer equally, depending on personality. Maan et al. (2020) pointed out that, under the basis of social exchange and fairness theory, employees with proactive personalities may have higher job satisfaction, better innovative work behavior, and stronger learning goal orientations. A multifaceted view of social exchange emphasizes the significance of many sources of support. According to these approaches, employees of proactive personalities develop different give-and-reward relationships under different organizational goals (Lavelle et al., 2007; Buil et al., 2019), so that they may generate stronger job satisfaction, deeper organizational commitment, and better in-role performance (Joo and Bennett III, 2018). Managers can help employees adapt to the work environment and have better job satisfaction by identifying and managing their motivations and opportunities. However, employees with proactive personality traits can also negatively affect their in-role performance, if they use emotional adjustment strategies at a superficial level. Nonetheless, the effect of deep acting on in-role performance is not obvious, which is different from what has been found by some previous researchers. The reason may be that the samples in this study are doctors and nurses in health institutions and hospitals. These individuals are professionals and are respected by people and have high salaries, all of which may cause the average person to behave well towards them.

In addition, in Chinese culture, there is a leader-centered power relationship network, so the leader’s partiality to insiders will make the employees feel that they are being treated fairly and being given corresponding powers with clear obligations in the invisible norms of the organizers in the circle. This could have a positive effect on their performance (Schaubroeck et al., 2017). Scholars refer to differential leadership as the leadership style in which a leader gives more partial treatment to their own subordinates. Although this leadership behavior may seem unfair, it is common in Chinese business organizations where the emphasis on human relations is rather common (Mingzheng and Xinhui, 2014; Li et al., 2017. In this study, however, the effect is not significant. Insiders may have a good relationship with the leader as mentioned above, but thus become arrogant. They may think their job condition is stable and not feel any need to work hard, thus resulting in poor in-role performance. For outsiders, if they are not treated in the same way as insiders, they may have the feeling of being treated unfavorably. This feeling has a high negative correlation with performance, job satisfaction, and loyalty (Jiang and Zhang, 2010). This finding is consistent with the hypothesis of this study. In facing this kind of prejudicial treatment, the feeling of injustice increases over time, which not only consumes the employees’ positive psychology, but also reduces the degree of their investment in the organization. Therefore, employees will be satisfied and motivated only when they perceive fairness. When they do, they are likely to continue to work actively. When unfairness is perceived, however, then, in order to compensate for that sense of injustice, employees may reduce work engagement and organizational commitment, and may also retaliate against the organization (Zhu and Xie, 2018). When insiders with proactive personality traits are treated favorably under differential leadership, the stronger their sense of organizational justice, the more engaged they are at work, and their in-role performance is significantly enhanced. On the other hand, outsiders with proactive personality traits, in the face of unfair interpersonal interactions, will look for ways to address their predicament and achieve their expected goals.

### Theoretical implications of the research

From a theoretical point of view, this study has the following contributions. First, it can be used as the basis for deepening differential leadership. This study finds that leaders’ differential treatment of different subordinates has group-level implications, and the results derived from differential treatment can reflect leaders’ differential treatment behavior. It is a response to Jiang and Zhang’s call (2010) to examine the relevance of differential leadership at different levels. Furthermore, this study agrees with both Jiang and Zhang (2010)’s and Leung and Barnes (2020)’s claims that leaders expect differential treatment to empower insiders so as to increase loyalty and effectiveness, and that subordinates outside the circle can also take the initiative to learn from their fellow workers and show behaviors that meet the expectations of their leaders.

Second, this research contributes to the research literature on the fairness of resource allocation, in regards to how fairness affects not only the individual rights of employees, but also the overall organizational performance. Differential leadership will therefore affect differential performance (Li et al., 2018). The results show that, with proactive personality traits, both “insiders” (who are treated favorably) and “outsiders” (who are treated unfavorably) may have a positive impact on organizational justice and on in-role performance – and if they succeed in this, it may lessen their negative perceptions of the organization. Our study is the first to simultaneously consider differential leadership bias (DLB) against “outsiders,” proactive personality, and organizational justice in a single study. The results suggest that the effects of flawed differential leadership can also be motivating, as long as managers pay more attention to understanding employee traits when applying a differential leadership strategy.

### Practical implications of the research

Whether the trusted subordinates can establish a harmonious and mutually beneficial relationship with other subordinates in the group is worthy of attention. If leaders can treat their subordinates fairly, regardless of whether they are inside or outside of their circles, and if they can promote mutual cooperation and assistance among all subordinates, then it may foster friendly relationships, both with subordinates and also between insiders and outsiders. Furthermore, through the two-way communication of insiders, the gap between a leader and the subordinates outside the circle (outsiders) can be reduced, and the subordinates outside the circle can also better understand the ideas and expectations of their leader, which may improve their chances of “upgrading” to insiders. In contrast, if a leader gives privileges to and tolerates mistakes from cronies based on personal relationships rather than their work performance, then these cronies may become arrogant and start bullying outsiders. Adding to anger and disapproval of cronies, outsiders will also have a negative impression on their leaders. Therefore, as Akram et al. (2021) has proposed, managers should provide opportunities for employees to participate and create a fair atmosphere in their organization, so as to mitigate the harmful consequences of abusive supervision.

In addition, this study confirms that outsiders with proactive personality traits also have an overall impact on both their own performance and also the performance of others on the inside, in the process of transformation. For employees outside the circle, getting help and support from insiders can reduce obstacles in the transition process, so as to optimize the allocation of high-quality resources by the controller. For insiders, new outsiders can be used as partners (Wang, 2015). Not only is it beneficial to their own work performance, but it also helps outsiders reduce the difficulties encountered in entering their circle within the organization. Both sides can benefit, and reduce the sense of threat to their status from the other (Fan and Zheng, 2000). Also, the sense of responsibility in the personality traits of medical staff is negatively correlated with surface acting, but is positively correlated with deep acting (Lu and Sun, 2021). This is likely because managers fully consider the emotional expression requirements of different post characteristics and thus choose medical staff with matching personality characteristics.

### Limitations and directions for future research

While this research provides theoretical and managerial implications, there are some limitations to be acknowledged. First, due to time constraints and the pandemic, we were not able to conduct an actual interview with the survey respondents. All the questionnaires were distributed and collected through the contacts at the hospital and health institutions. Second, this study only investigated hospital staff, so the results may be consequently biased. It is not known what situation the respondents were in when they filled out the questionnaire, and there were difficulties in determining whether they were affected by constraints of the external environment. Third, this study selected medical staff from hospitals in the six major urban areas of Beijing, from Grade 1 to Grade 3-and-above. Thus, whether these survey results can also be inferred to employees in different regions and in different industries needs further verification. Fourth, there is a limitation of research dimensions. All variables in this study contain multiple dimensional structures and we did not explore the other variable dimensions or explore the mutual influence relationships among them, in a one-by-one manner.

There are several avenues for future studies. First, the research object can be expanded in scope or targeted to the service sector or to other related sectors. It is possible to further explore the impact of employees’ own feelings and interactions on personal effectiveness. Alternatively, new research on the relationship caused by the differential atmosphere from different perspectives can be analyzed by paired samples. Second, in a differential leadership style, it is possible to further explore topics such as authoritarian and paternalistic leaders’ differential treatment of employees based on tasks and emotions, which affects employee classification and self-perceived classification, as well as their active insider-and-outsider transfer process. Third, we can further explore the positive role of proactive personality and differential leadership styles in differential management areas, its impact on the effectiveness of the overall team, and the relationship conflicts between teams. Fourth, in a relational atmosphere supported by emotional commitment, the mediating relationship between emotional labor and differential management behavior in the team should be explored, and the relationship between its internal influence and the matching environment in the individual-organization should also be discussed. Fifth, in order to explore the overall impact, future scholars can discuss these variables in detail, so as to enhance the understanding of the influence of these factors. Follow-up researchers can consider the research issues and environmental constraints, and select objects and methods that are suitable for their own research and development conclusions.

## Data availability statement

The original contributions presented in the study are included in the article/Supplementary material, further inquiries can be directed to the corresponding author.

## Ethics statement

Ethical review and approval was not required for the study on human participants in accordance with the local legislation and institutional requirements. Written informed consent from the participants was not required to participate in this study in accordance with the national legislation and the institutional requirements.

## Author contributions

S-TC was mainly responsible for writing the manuscript and analyzing the data. KH was in charge of revising and improving the manuscript.

## Conflict of interest

The authors declare that the research was conducted in the absence of any commercial or financial relationships that could be construed as a potential conflict of interest.

## Publisher’s note

All claims expressed in this article are solely those of the authors and do not necessarily represent those of their affiliated organizations, or those of the publisher, the editors and the reviewers. Any product that may be evaluated in this article, or claim that may be made by its manufacturer, is not guaranteed or endorsed by the publisher.

## Supplementary material

The Supplementary material for this article can be found online at: https://www.frontiersin.org/articles/10.3389/fpsyg.2022.978495/full#supplementary-material

## References

[ref1] Aarons G. A. Ehrhart M. G. Farahnak L. R. Sklar M. Horowitz J. (2017). Discrepancies in leader and follower ratings of transformational leadership: relationship with organizational culture in mental health. Adm. Policy Ment. Health Ment. Health Serv. Res. 44, 480–491. doi: 10.1007/s10488-015-0672-7, PMID: 26164567 PMC4775440

[ref001] Adams J. S. (1965). “Inequity in social exchange,” in Advances in Experimental Psychology. ed. Berkowitz L. New York: Academic Press.

[ref2] Aiken L. S. West S. G. Reno R. R. (1991). Multiple Regression: Testing and Interpreting Interactions. London: Sage.

[ref3] Akram Z. Ahmad S. Akram U. Asghar M. Jiang T. (2021). Is abusive supervision always harmful toward creativity? Managing workplace stressors by promoting distributive and procedural justice. Int. J. Confl. Manag. 33, 385–407. doi: 10.1108/IJCMA-03-2021-0036

[ref4] Albalawi A. S. Naughton S. Elayan M. B. Sleimi M. T. (2019). Perceived organizational support, alternative job opportunity, organizational commitment, job satisfaction and turnover intention: a moderated-mediated model. Organ 52, 310–324. doi: 10.2478/orga-2019-0019

[ref5] Al-Omar H. A. Arafah A. M. Barakat J. M. Almutairi R. D. Khurshid F. Alsultan M. S. (2019). The impact of perceived organizational support and resilience on pharmacists’ engagement in their stressful and competitive workplaces in Saudi Arabia. Saudi Pharm. J. 27, 1044–1052. doi: 10.1016/j.jsps.2019.08.007, PMID: 31997912 PMC6978622

[ref6] Anderson J. C. Gerbing D. W. (1988). Structural equation modeling in practice: a review and recommended two-step approach. Psychol. Bull. 103, 411–423. doi: 10.1037/0033-2909.103.3.411

[ref7] Ashforth B. E. Humphrey R. H. (1993). Emotional labor in service roles: the influence of identity. Acad. Manag. Rev. 18, 88–115. doi: 10.2307/258824

[ref8] Asrar-ul-Haq M. Kuchinke K. P. (2016). Impact of leadership styles on employees’ attitude towards their leader and performance: empirical evidence from Pakistani banks. Future Bus. J. 2, 54–64. doi: 10.1016/j.fbj.2016.05.002

[ref9] Bateman T. S. Crant J. M. (1993). The proactive component of organizational behavior: a measure and correlates. J. Organ. Behav. 14, 103–118. doi: 10.1002/job.4030140202

[ref10] Blakely G. L. Srivastava A. Moorman R. H. (2005). The effects of nationality，work role centrality, and work locus of control on role definitions of OCB. J. Leadersh. Org. Stud., 12, 103–117. doi: 10.1177/107179190501200109

[ref11] Buil I. Martínez E. Matute J. (2019). Transformational leadership and employee performance: the role of identification, engagement and proactive personality. Int. J. Hosp. Manag. 77, 64–75. doi: 10.1016/j.ijhm.2018.06.014

[ref12] Bulathsinhalage S. Pathirawasam C. (2017). The effect of corporate governance on firms’ capital structure of listed companies in Sri Lanka. J. Comp. 9, 19–33. doi: 10.7441/joc.2017.02.02

[ref13] Burns A. J. Roberts T. L. Posey C. Lowry P. B. (2019). The adaptive roles of positive and negative emotions in organizational insiders’ security-based precaution taking. Inf. Syst. Res. 30, 1228–1247. doi: 10.1287/isre.2019.0860

[ref14] Chapman B. P. Goldberg L. R. (2017). Act-frequency signatures of the big five. Personal. Individ. Differ. 116, 201–205. doi: 10.1016/j.paid.2017.04.049, PMID: 29379221 PMC5785942

[ref15] Cheng B. S. Farh J. L. Cheng H. F. Hsu W. L. (2002). Guanxi, Zhong cheng, competence and managerial behavior in the Chinese context, journal of Chinese. Psychology 44, 151–162.

[ref16] Cheng P.-H. Lin J.-W. (1998). The differential patterns and organizational behavior of Chinese behavior: a preliminary study of large private Enterprises in Taiwan. Central Inst. Ethn. Stud. 86, 29–72.

[ref17] Crant J. M. (2000). Proactive behavior in organizations. J. Manag. 26, 435–462. doi: 10.1177/014920630002600304

[ref18] Cropanzano R. Mitchell M. S. (2005). Social exchange theory: an interdisciplinary review. J. Manag. 31, 874–900. doi: 10.1177/0149206305279602

[ref20] De Hoogh A. H. Greer L. L. Den Hartog D. N. (2015). Diabolical dictators or capable commanders? An investigation of the differential effects of autocratic leadership on team performance. Leadersh. Q. 26, 687–701. doi: 10.1016/j.leaqua.2015.01.001

[ref21] Delgado C. Upton D. Ranse K. Furness T. Foster K. (2017). Nurses’ resilience and the emotional labour of nursing work: an integrative review of empirical literature. Int. J. Nurs. Stud. 70, 71–88. doi: 10.1016/j.ijnurstu.2017.02.008, PMID: 28235694

[ref002] Diefendorff J. M. Richard E. M. Croyle M. H. (2006). Are emotional display rules formal job requirements? Examination of employee and supervisor perceptions. J. Occup. Psychol. 79, 273–298. doi: 10.1348/096317905X68484

[ref22] Ding M. J. Jie F. (2021). The moderating effect of Guanxi on supply chain competencies of logistics firms in China. Int J Log Res Appl 24, 407–425. doi: 10.1080/13675567.2020.1763280

[ref23] Donia M. B. Raja U. Panaccio A. Wang Z. (2016). Servant leadership and employee outcomes: the moderating role of subordinates’ motives. Eur. J. Work Organ. Psy. 25, 722–734. doi: 10.1080/1359432X.2016.1149471

[ref24] Durana P. Perkins N. Valaskova K. (2021). Artificial intelligence data-driven internet of things systems, real-time advanced analytics, and cyber-physical production networks in sustainable smart manufacturing. Econ. Manag. Finan. Mark. 16, 20–31. doi: 10.22381/emfm16120212

[ref003] Eisenberger R. Karagonlar G. Stinglhamber F. Neves P. Becker T. E. Gonzalez-Morales M. G.. (2010). Leader–member exchange and affective organizational commitment: The contribution of supervisor’s organizational embodiment. J. Appl. Soc. Psychol. 95, 1085–1103. doi: 10.1037/a0020858 20718516

[ref25] Engelbrecht A. Samuel O. M. (2019). The effect of transformational leadership on intention to quit through perceived organisational support, organisational justice and trust. S. Afr. J. Econ. Manag. Sci. 22, 1–8. doi: 10.4102/sajems.v22i1.2338

[ref004] Epitropaki O. Kapoutsis I. Ellen III B. P. Ferris G. R. Drivas K. Ntotsi A. (2016). Navigating uneven terrain: the roles of political skill and LMX differentiation in prediction of work relationship quality and work outcomes. J. Organ. Behav. 37, 1078–1103. doi: 10.1002/job.2100

[ref26] Erdogan B. Enders J. (2007). Support from the top: supervisors' perceived organizational support as a moderator of leader-member exchange to satisfaction and performance relationships. J. Appl. Psychol. 92, 321–330. doi: 10.1037/0021-9010.92.2.321, PMID: 17371081

[ref27] Erez A. Isen A. M. (2002). The influence of positive affect on components of expectancy motivation. J. Appl. Psychol. 87, 1055–1067. doi: 10.1037/0021-9010.87.6.1055 12558213

[ref28] Fan J.-L. Zheng B. O. (2000). Paternalistic leadership in Chinese organizations: an analysis of a cultural perspective. Native Psychol. Res. 13, 127–180. doi: 10.1017/CBO9780511753763.008

[ref29] Farahnak L. R. Ehrhart M. G. Torres E. M. Aarons G. A. (2020). The influence of transformational leadership and leader attitudes on subordinate attitudes and implementation success. J. Leadersh. Organ. Stud. 27, 98–111. doi: 10.1177/1548051818824529

[ref30] Feng L. Li J. Feng T. Jiang W. (2019). Workplace ostracism and job performance: meaning at work and family support as moderators. Soc. Behav. Personal. Int. J. 47, 1–13. doi: 10.2224/sbp.8244

[ref31] Fornell C. Larcker D. F. (1981). Evaluating structural equation models with unobservable variables and measurement error. J. Mark. Res. 18, 39–50. doi: 10.1177/002224378101800104

[ref32] Gefen D. Straub D. Boudreau M. C. (2000). Structural equation modeling and regression: guidelines for research practice. Commun. Assoc. Inf. Syst. 4:7. doi: 10.17705/1CAIS.00407

[ref33] Ghosh D. Sekiguchi T. Gurunathan L. (2017). Organizational embeddedness as a mediator between justice and in-role performance. J. Bus. Res. 75, 130–137. doi: 10.1016/j.jbusres.2017.02.013

[ref005] Ghiselli E. E. Campbell J. P. Zedeck S. (1981). Measurement theory for the behavioral sciences. W.H. Freeman.

[ref34] Goldberg L. S. Grandey A. A. (2007). Display rules versus display autonomy: emotion regulation, emotional exhaustion, and task performance in a call center simulation. J. Occup. Health Psychol. 12, 301–318. doi: 10.1037/1076-8998.12.3.301, PMID: 17638495

[ref35] Grandey A. A. (2003). When“the show must go on”: surface acting and deep acting as determinants of emotional exhaustion and peer-ratedservice delivery. Acad. Manag. J. 46, 86–96. doi: 10.5465/30040678

[ref36] Harman H. H. (1976). Modern Factor Analysis. Chicago: University of Chicago Press.

[ref37] Hayes A. F. (2009). Beyond baron and Kenny: statistical mediation analysis in the new millennium. Commun. Monogr. 76, 408–420. doi: 10.1080/03637750903310360

[ref38] Hayes A. F. (2012). PROCESS: a versatile computational tool for observed variable mediation, moderation, and conditional process modeling [white paper]. Available at: http://www.afhayes.com/public/process2012.pdf

[ref39] Hayyat U. Nisar Q. A. Imran M. Ikram M. (2017). Consequences of emotional labor in health sector of Pakistan. Int. J. Res. Bus. Manag. Account. 3, 64–79. doi: 10.53555/bma.v3i3.1714

[ref41] Homans G. C. (1958). Social behavior as exchange. Am. J. Sociol. 63, 597–606. doi: 10.1086/222355

[ref42] Jahanzeb S. Fatima T. Javed B. Giles J. P. (2020). Can mindfulness overcome the effects of workplace ostracism on job performance? J. Soc. Psychol. 160, 589–602. doi: 10.1080/00224545.2019.1707465, PMID: 31870244

[ref43] Jha B. Kumar A. (2016). Employee engagement: a strategic tool to enhance performance. DAWN J. Contem. Res. Manag. 3, 21–29.

[ref44] Jiang D.-Y. Cheng B.-H. (2014). The nature of Chinese differential leadership and the process of shadowing. Stud. Native Psychol. 42, 285–357.

[ref45] Jiang D.-Y. Zhang I. Z. (2010). Chinese differential leadership and ministry effectiveness. Native Psychol. Res. 33, 109–177.

[ref46] Joo B. K. B. Bennett R. H. (2018). The influence of proactivity on creative behavior, organizational commitment, and job performance: evidence from a Korean multinational. J. Int. Interdis. Bus. Res. 5, 1–20.

[ref47] Jöreskong K. G. Sörbom D. (1998). LISREL 8: Structural Equation Modelling with the SIMPLIS Command Language. Chicago: Scientific Software International.

[ref48] Judge T. A. Zapata C. P. (2015). The person–situation debate revisited: effect of situation strength and trait activation on the validity of the big five personality traits in predicting job performance. Acad. Manag. J. 58, 1149–1179. doi: 10.5465/amj.2010.0837

[ref49] Kang D. Y. Cheung I. C. (2010). Chinese differential leadership and subordinate effectiveness. Res. Native Psychol. 33, 109–177.

[ref50] Keskes I. Sallan J. M. Simo P. Fernandez V. (2018). Transformational leadership and organizational commitment: mediating role of leader-member exchange. J. Manag. Dev. 37, 271–284. doi: 10.1108/JMD-04-2017-0132

[ref51] Kliestik T. Nica E. Musa H. Poliak M. Mihai E. A. (2020). Networked, smart, and responsive devices in industry 4.0 manufacturing systems. Econ. Manag. Finan. Mark. 15, 23–30. doi: 10.22381/EMFM15320203

[ref52] Krulicky T. Horak J. (2021). Business performance and financial health assessment through artificial intelligence. Ekonomicko-manazerske spektrum 15, 38–51. doi: 10.26552/ems.2021.2.38-51

[ref53] Lavelle J. J. Rupp D. E. Brockner J. (2007). Taking a multifoci approach to the study of justice, social exchange, and citizenship behavior: the target similarity model. J. Manag. 33, 841–866. doi: 10.1177/0149206307307635

[ref54] Lazaroiu G. Kliestik T. Novak A. (2021). Internet of things smart devices, industrial artificial intelligence, and real-time sensor networks in sustainable cyber-physical production systems, journal of self-governance and management. Economics 9, 20–30.

[ref55] Leung T. K. P. Barnes B. R. (2020). Ethical cronyism: an insider approach for building guanxi and leveraging business performance in China. Asia Pac. Bus. Rev. 26, 124–148. doi: 10.1080/13602381.2019.1654215

[ref56] Li X. Dang G. Gao D. (2018). The effect of differential leadership on turnover intention: the role of proactive personality and workplace ostracism. Psychol. Res. 11, 444–451. doi: 10.3969/j.issn.2095-1159.2018.05.007

[ref57] Li S. L. Huo Y. Long L. R. (2017). Chinese traditionality matters: effects of differentiated empowering leadership on followers’ trust in leaders and work outcomes. J. Bus. Ethics 145, 81–93. doi: 10.1007/s10551-015-2900-1

[ref59] Lu S. Sun X.-L. (2021). Advances in research on emotional labor management of nurses. Chin. J. Nurs. 56:6. doi: 10.3761/j.issn.0254-1769.2021.01.023

[ref60] Luo J. D. Cheng M. Y. Zhang T. (2016). Guanxi circle and organizational citizenship behavior: context of a Chinese workplace. Asia Pac. J. Manag. 33, 649–671. doi: 10.1007/s10490-016-9479-7

[ref61] Maan A. T. Abid G. Butt T. H. Ashfaq F. Ahmed S. (2020). Perceived organizational support and job satisfaction: a moderated mediation model of proactive personality and psychological empowerment. Future Bus. J. 6, 1–12. doi: 10.1186/s43093-020-00027-8

[ref62] Matschke C. Fehr J. (2015). Internal motivation buffers the negative effect of identity incompatibility on new comers’ social identification and well-being. Soc. Psychol. 46, 335–344. doi: 10.1027/1864-9335/a000250

[ref63] McDonald R. P. Ho M. H. R. (2002). Principles and practice in reporting structural equation analyses. Psychol. Methods 7, 64–82. doi: 10.1037/1082-989X, PMID: 11928891

[ref64] Miller E. R. Gkonou C. (2018). Language teacher agency, emotion labor and emotional rewards in tertiary-level English language programs. System 79, 49–59. doi: 10.1016/j.system.2018.03.002

[ref65] Mingzheng X. Xinhui W. (2014). Chinese leadership: culture and confucianism. Public Integ. 16, 165–172. doi: 10.2753/PIN1099-9922160204

[ref66] Mitan A. Siekelova A. Rusu M. Rovnak M. (2021). Value-based management: a case study of visegrad four countries. Ekonomicko-manazerske spektrum 15, 87–98. doi: 10.26552/ems.2021.2.87-98

[ref67] Morris J. A. Feldman D. C. (1996). The dimensions, antecedents and consequences of emotional labor. Acad. Manag. Rev. 21, 986–1010. doi: 10.2307/259161

[ref68] Muthukrishnan R. Hansel-Welch N. Larkin D. J. (2018). Environmental filtering and competitive exclusion drive biodiversity-invasibility relationships in shallow lake plant communities. J. Ecol. 106, 2058–2070. doi: 10.1111/1365-2745.12963

[ref69] Nauman S. Zheng C. Naseer S. (2020). Job insecurity and work–family conflict: a moderated mediation model of perceived organizational justice, emotional exhaustion and work withdrawal. Int. J. Confl. Manag. 31, 729–751. doi: 10.1108/IJCMA-09-2019-0159

[ref70] Newman A. Schwarz G. Cooper B. Sendjaya S. (2017). How servant leadership influences organizational citizenship behavior: the roles of LMX, empowerment, and proactive personality. J. Bus. Ethics 145, 49–62. doi: 10.1007/s10551-015-2827-6

[ref71] Niehoff B. P. Moorman R. H. (1993). Justice as a mediator of the relationship between methods of monitoring and organizational citizenship behavior. Acad. Manag. J. 36, 527–556. doi: 10.2307/256591

[ref72] Nunnally J. C. Bernstein I. H. (1994). Psychometric Theory. New York: McGraw Hill.

[ref73] Persson P. Zhuravskaya E. (2016). The limits of career concerns in federalism: evidence from China. J. Eur. Econ. Assoc. 14, 338–374. doi: 10.1111/jeea.12142

[ref74] Phuoc N. H. Hau L. N. Thuy P. N. (2022). The dual outcomes of frontliner’s autonomous motivation and deep acting in service co-creation: a dyadic approach. Serv. Bus. 16, 159–186. doi: 10.1007/s11628-021-00473-6

[ref75] Piansoongnern O. (2016). Chinese leadership and its impacts on innovative work behavior of the Thai employees. Glob. J. Flex. Syst. Manag. 17, 15–27. doi: 10.1007/s40171-015-0110-4

[ref77] Pournader M. Kach A. Talluri S. (2020). A review of the existing and emerging topics in the supply chain risk management literature. Decis. Sci. 51, 867–919. doi: 10.1111/deci.12470, PMID: 34234385 PMC7283689

[ref78] Probst T. M. Gailey N. J. Jiang L. Bohle S. L. (2017). Psychological capital: buffering the longitudinal curvilinear effects of job insecurity on performance. Saf. Sci. 100, 74–82. doi: 10.1016/j.ssci.2017.02.002

[ref79] Raja U. Johns G. (2010). The joint effects of personality and job scope on in-role performance, citizenship behaviors, and creativity. Hum. Relat. 63, 981–1005. doi: 10.1177/0018726709349863

[ref80] Ren J. Lin M. C. Chen E. H. (2002). Criteria and empirical study of Chinese employee categorization. in Proceedings of the Fourth Symposium of Chinese Psychologists, Taipei.

[ref81] Roberts Z. Rogers A. Thomas C. L. Spitzmueller C. (2018). Effects of proactive personality and conscientiousness on training motivation. Int. J. Train. Dev. 22, 126–143. doi: 10.1111/ijtd.12122

[ref82] Rodrigues N. Rebelo T. (2019). Predicting innovative performance through proactive personality: examining its criterion validity and incremental validity over the five-factor model. Int. J. Sel. Assess. 27, 1–8. doi: 10.1111/ijsa.12232

[ref84] Salas-Vallina A. Alegre J. López-Cabrales Á. (2021). The challenge of increasing employees' well-being and performance: how human resource management practices and engaging leadership work together toward reaching this goal. Hum. Resour. Manag. 60, 333–347. doi: 10.1002/hrm.22021

[ref85] Schaubroeck J. M. Shen Y. Chong S. (2017). A dual-stage moderated mediation model linking authoritarian leadership to follower outcomes. J. Appl. Psychol. 102, 203–214. doi: 10.1037/apl0000165, PMID: 27786498

[ref86] Schreuder E. Van Erp J. Toet A. Kallen V. L. (2016). Emotional responses to multisensory environmental stimuli: a conceptual framework and literature review. SAGE Open 6:215824401663059. doi: 10.1177/2158244016630591

[ref88] Sexton J. B. Adair K. C. Leonard M. W. Frankel T. C. Proulx J. Watson S. R.. (2018). Providing feedback following leadership WalkRounds is associated with better patient safety culture, higher employee engagement and lower burnout. BMJ Qual. Safety 27, 261–270. doi: 10.1136/bmjqs-2016-006399, PMID: 28993441 PMC5867443

[ref89] Shu C. Y. Lazatkhan J. (2017). Effect of leader-member exchange on employee envy and work behavior moderated by self-esteem and neuroticism. Revista de Psicología del Trabajo y de las Organizaciones 33, 69–81. doi: 10.1016/j.rpto.2016.12.002

[ref90] Tijani A. A. Osagie R. O. Afolabi K. B. (2021). Effect of strategic alliance and partnership on the survival of MSMEs post COVID-19 pandemic. Ekonomicko-manazerske spektrum 15, 126–137. doi: 10.26552/ems.2021.2.126-137

[ref91] Turban D. B. Moake T. R. Wu S. Y. H. Cheung Y. H. (2017). Linking extroversion and proactive personality to career success: the role of mentoring received and knowledge. J. Career Dev. 44, 20–33. doi: 10.1177/0894845316633788

[ref92] Valaskova K. Ward P. Svabova L. (2021). Deep learning-assisted smart process planning, cognitive automation, and industrial big data analytics in sustainable cyber-physical production systems. J. Self-Govern. Manag. Econ. 9, 9–20. doi: 10.22381/jsme9220211

[ref006] Van de Vijver F. Leung K. (1997). “Methods and data analysis of comparative research,” in Handbook of cross-cultural psychology. 257–300.

[ref93] Vermooten N. Boonzaier B. Kidd M. (2019). Job crafting, proactive personality and meaningful work: implications for employee engagement and turnover intention. SA J. Ind. Psychol. 45, 1–13. doi: 10.4102/sajip.v45i0.1567

[ref94] Vigoda E. (2000). Internal politics in public administration systems: an empirical examination of its relationship with job congruence, organizational citizenship behavior, and in-role performance. Public Pers. Manag. 29, 185–210. doi: 10.1177/009102600002900203

[ref95] Wang X. Y. (2009). An empirical analysis of the structure and status of employees’ perceptions of organizational equity in China. Manag. Rev. 21, 39–47. doi: 10.4236/jhrss.2015.34022

[ref96] Wang L. (2015). The effects of differential leadership on employee and team creativity in growing Chinese family firms: a cross-level follow-up study. Adv. Psychol. Sci. 23, 1688–1700. doi: 10.3724/SP.J.1042.2015.01688

[ref97] Wang T. Cao Z. Zhong X. Chen C. (2021). Self-regulation failure? The influence mechanism of leader reward omission on employee deviant behavior. Front. Psychol. 12, 1–12. doi: 10.1002/job.85 PMC8058438 33897510

[ref98] Wang H. Guan B. (2018). The positive effect of authoritarian leadership on employee performance: the moderating role of power distance. Front. Psychol. 9:357. doi: 10.3389/fpsyg.2018.00357, PMID: 29628902 PMC5876282

[ref99] Wang H. Lei L. (2021). Proactive personality and job satisfaction: social support and Hope as mediators. Curr. Psychol., 1–10. doi: 10.1007/s12144-021-01379-2

[ref100] Wang L. Li L. Z. (2011). Advances in research on nurses’ emotional labor. Chin. J. Nurs. 46, 314–316.

[ref102] Wen J. Huang S. S. Hou P. (2019). Emotional intelligence, emotional labor, perceived organizational support, and job satisfaction: a moderated mediation model. Int. J. Hosp. Manag. 81, 120–130. doi: 10.1016/j.ijhm.2019.01.009

[ref103] Xiong Chen Z. Aryee S. (2007). Delegation and employee work outcomes: an examination of the cultural context of mediating processes in China. Acad. Manag. J. 50, 226–238. doi: 10.5465/amj.2007.24162389

[ref104] Xu J. Liu F. (2020). The impact of intellectual capital on firm performance: a modified and extended VAIC model. J. Compet. 12, 161–176. doi: 10.7441/joc.2020.01.10

[ref106] Yang F. Chau R. (2016). Proactive personality and career success. J. Manag. Psychol. 31, 467–482. doi: 10.1108/JMP-04-2014-0139

[ref107] Yang L. Jiang D. Sahli H. (2018). Integrating deep and shallow models for multi-modal depression analysis—hybrid architectures. IEEE Trans. Affect. Comput. 12, 239–253. doi: 10.1109/TAFFC.2018.2870398

[ref108] Yean T. F. (2016). Organizational justice: A conceptual discussion. Procedia Soc. Behav. Sci. 219, 798–803. doi: 10.1016/j.sbspro.2016.05.082

[ref109] Yin Z. Zhao M. Wang Y. Yang J. Zhang J. (2017). Recognition of emotions using multimodal physiological signals and an ensemble deep learning model. Comput. Methods Prog. Biomed. 140, 93–110. doi: 10.1016/j.cmpb.2016.12.005, PMID: 28254094

[ref110] Zheng B. O. (1995). Differential order patterns and Chinese organizational behavior. Res. Native Psychol. 2, 142–219.

[ref111] Zheng O. B. (2006). Differential leaderships and Chinese organizational behavior. Chin. Sci. Psychol. Rev. 2

[ref112] Zhu Y. Xie B. B. (2018). The relationship between the perceived climate of team cha-xu and employee silence: research on affective commitment and traditionality. J. Psychol. 50, 539–548. doi: 10.3724/SP.J.1041.2018.00539

