# Supplementary material for: The influence of differential leadership and proactive personality on employee in-role performance: An integrated model
Source: Front Psychol. 2022 Dec 22;13:978495. doi: 10.3389/fpsyg.2022.978495 (PMC9815459; doi:10.3389/fpsyg.2022.978495)
Supplement: Supplementary file 1 [file Data_Sheet_1.docx]

**Appendix A**

Process syntax (moderating effect)

process y=IRP/x=pp/m=OJ/w=ELSP/z=ELDP/cov=PPxDLP PPxDLB DLP DLB/total=1/cmatrix=1,1,1,1,0,0,1,1 / bmatrix=1,1,1/ wmatrix=0,1,0/ zmatrix=0,1,0.

Process syntax (indirect effect)

process y=IRP/x=pp/m=OJ/cov=PPxDLP PPxDLB DLP DLB/total=1/normal=1 /decimals=F10.3/model=4/cmatrix=1,1,1,1,0,0,1,1 .

process y=IRP/x=PPxDLP/m=OJ/cov=pp PPxDLB DLP DLB/total=1/normal=1 /decimals=F10.3/bmatrix=1,0,1/cmatrix=1,1,1,1,1,0,1,1 .

process y=IRP/x=PPxDLB/m=OJ/cov=pp PPxDLP DLP DLB/total=1/normal=1 /decimals=F10.3/bmatrix=1,0,1/cmatrix=1,1,1,1,1,0,1,1 .

process y=IRP/x=DLP/m=OJ/cov=pp PPxDLP PPxDLB DLB/total=1/normal=1 /decimals=F10.3/model=4/cmatrix=1,1,1,1,1,0,0,1 .

process y=IRP/x=DLB/m=OJ/cov=pp PPxDLP PPxDLB DLP /total=1/normal=1 /decimals=F10.3/model=4/cmatrix=1,1,1,1,1,0,0,1 .

**Appendix B**

**Table 2: Frequency table**

|  | | Frequency | Percent | Valid Percent | Cumulative Percent |
| --- | --- | --- | --- | --- | --- |
| **Sex** | 1 male | 167 | 31.9 | 31.9 | 31.9 |
|  | 2 female | 357 | 68.1 | 68.1 | 100.0 |
|  | Total | 524 | 100.0 | 100.0 |  |
| **age** | 1 below 20 | 59 | 11.3 | 11.3 | 11.3 |
|  | 2 21-30 | 313 | 59.7 | 59.7 | 71.0 |
|  | 3 31-40 | 112 | 21.4 | 21.4 | 92.4 |
|  | 4 41-50 | 40 | 7.6 | 7.6 | 100.0 |
|  | Total | 524 | 100.0 | 100.0 |  |
| **edu** | 1 technical school | 52 | 9.9 | 9.9 | 9.9 |
|  | 2 Junior college | 149 | 28.4 | 28.4 | 38.4 |
|  | 3 undergraduate | 252 | 48.1 | 48.1 | 86.5 |
|  | 4 master | 63 | 12 | 12 | 98.5 |
|  | 5 phd above | 8 | 1.5 | 1.5 | 100 |
|  | Total | 524 | 100 | 100 |  |
| **senior** | 1 1 year | 56 | 10.7 | 10.7 | 10.7 |
|  | 2 2-5 years | 256 | 48.9 | 48.9 | 59.5 |
|  | 3 5-10 years | 145 | 27.7 | 27.7 | 87.2 |
|  | 4 10-20 years | 40 | 7.6 | 7.6 | 94.8 |
|  | 5 above 20 years | 27 | 5.2 | 5.2 | 100.0 |
|  | Total | 524 | 100.0 | 100.0 |  |
| **level** | 1 none | 325 | 62.0 | 62.0 | 62.0 |
|  | 2 basic | 134 | 25.6 | 25.6 | 87.6 |
|  | 3 middle class | 48 | 9.2 | 9.2 | 96.8 |
|  | 4 high class | 17 | 3.2 | 3.2 | 100.0 |
|  | Total | 524 | 100.0 | 100.0 |  |
| **scale** | 1 below first degree | 51 | 9.7 | 9.7 | 9.7 |
|  | 2 secondary degree | 218 | 41.6 | 41.6 | 51.3 |
|  | 3 above third degree | 255 | 48.7 | 48.7 | 100.0 |
|  | Total | 524 | 100.0 | 100.0 |  |
